# Taking a closer look: disentangling effects of functional diversity on ecosystem functions with a trait-based model across hierarchy and time

**DOI:** 10.1098/rsos.140541

**Published:** 2015-03-25

**Authors:** Frédéric Holzwarth, Nadja Rüger, Christian Wirth

**Affiliations:** 1 AG Spezielle Botanik und Funktionelle Biodiversität, Institut für Biologie, Universität Leipzig, Johannisallee 21, 04103 Leipzig, Germany; 2 German Centre for Integrative Biodiversity Research (iDiv) Halle-Jena-Leipzig, Deutscher Platz 5e, 04103 Leipzig, Germany; 3 Smithsonian Tropical Research Institute, Apartado 0843-03092, Balboa Ancón, Panama

**Keywords:** biodiversity and ecosystem functioning, functional composition, hierarchical trait influence, forest succession, trait-based modelling, LPJ-GUESS

## Abstract

Biodiversity and ecosystem functioning (BEF) research has progressed from the detection of relationships to elucidating their drivers and underlying mechanisms. In this context, replacing taxonomic predictors by trait-based measures of functional composition (FC)—bridging functions of species and of ecosystems—is a widely used approach. The inherent challenge of trait-based approaches is the multi-faceted, dynamic and hierarchical nature of trait influence: (i) traits may act via different facets of their distribution in a community, (ii) their influence may change over time and (iii) traits may influence processes at different levels of the natural hierarchy of organization. Here, we made use of the forest ecosystem model ‘LPJ-GUESS’ parametrized with empirical trait data, which creates output of individual performance, community assembly, stand-level states and processes. To address the three challenges, we resolved the dynamics of the top-level ecosystem function ‘annual biomass change’ hierarchically into its various component processes (growth, leaf and root turnover, recruitment and mortality) and states (stand structures, water stress) and traced the influence of different facets of FC along this hierarchy in a path analysis. We found an independent influence of functional richness, dissimilarity and identity on ecosystem states and processes and hence biomass change. Biodiversity effects were only positive during early succession and later turned negative. Unexpectedly, resource acquisition (growth, recruitment) and conservation (mortality, turnover) played an equally important role throughout the succession. These results add to a mechanistic understanding of biodiversity effects and place a caveat on simplistic approaches omitting hierarchical levels when analysing BEF relationships. They support the view that BEF relationships experience dramatic shifts over successional time that should be acknowledged in mechanistic theories.

## 2. Introduction

Wood production and carbon storage by forests are of primary concern among the ecosystem services provided by terrestrial ecosystems. Forests store a large fraction and act as sources and sinks of significant amounts of carbon [1,2]. At the same time, the world's forests host nearly 100 000 tree species and tree diversity is a key feature of many tropical and temperate forest ecosystems [3]. Globally, tree species richness is declining due to, for example, the expansion of plantations or deforestation [4,5]. With this dramatic loss of species diversity, it is important to know how and to what extent plant diversity matters for the functioning of terrestrial ecosystems, especially the essential services of productivity and carbon storage [6,7].

Relationships between biodiversity and ecosystem functioning (BEF) have attracted increasing scientific interest since the seminal book of Schulze & Mooney in 1994 [8]. Significant and mostly positive effects of various facets of biodiversity on a wide range of ecosystem functions have been reported from grasslands experiments [7,9] and to some extent from forests [10,11]. For forests, however, experiments are far fewer and still young, thus their results are restricted to the very early and transient stages of a succession (‘BIOTREE’, since 2004: Scherer-Lorenzen *et al.* [12], ‘BEF China’, since 2009: Bruelheide *et al.* [13], since 2001: Potvin *et al.* [14]).

Since the early days of BEF research, science has progressed from the detection of relationships to elucidating their drivers and underlying mechanisms [15–18]. A promising approach to relate biodiversity to ecosystem functioning and to gain a mechanistic understanding is to focus on the diversity and identity of species traits relevant for the processes and functions under study [19]. In such a trait-based approach, taxonomic descriptors of biodiversity are replaced by measures of *functional composition* (FC) [20,21]. The challenge, often ignored when relating ecosystem functions to measures of FC, is the multi-faceted, dynamic and hierarchical nature of trait influence: (i) traits may function via different facets of their composition in a community, (ii) their influence may change over time and, most importantly, (iii) traits may influence processes at different levels of the natural hierarchy of organization.

*Multi-faceted nature of trait influence: Functional dissimilarity* (functional diversity *sensu stricto*, e.g. Petchey & Gaston [22]) reflects the trait dispersion within a community. Possible positive effects of higher functional dissimilarity on productivity and biomass accumulation in forests may arise from complementary use of light, water and nutrients [16,23], when ensembles of tree species exhibit a range of traits that allows sustained coexistence and a more efficient use of resources. *Functional identity*, the community weighted mean of trait values, reflects the average trait composition of the community [21,24]. Functional identity has been identified as a key component through which plant traits affect ecosystem functions [25–27], which is in line with Grime's mass ratio hypothesis [28]. *Functional richness* is defined by the trait extremes and thus reflects the potential shift of functional identity as well as the potential maximum functional dissimilarity. It defines the functional potential of a community, and as such is also a functional representation of the selection effect in that the higher the functional richness the higher the probability of including a dominant species [20].

*Dynamic nature of trait influence:* The role traits play for forest dynamics varies with ‘supply and demand’. Successional stages with high functional diversity offer more potential for complementary resource use, while those with low functional diversity and thus well-defined functional identity are likely to carry a stronger signature of identity effects on ecosystem functioning (cf. ‘shifting trait hypothesis’ *sensu* Wirth & Lichstein [29]). In the course of succession, resource availability (e.g. light, water) or the importance of demographic processes (e.g. growth, mortality and recruitment) may change. This is reflected by changes in the demand for particular trait configurations. In early successional stages, forest productivity is typically promoted by traits underlying an acquisitive strategy, whereas during later stages, trait expressions conveying adaptations to resource limitation become decisive [30]. While growth-related traits are important during the pioneer stage, mortality-related traits become relevant during the self-thinning and old-growth stages. As a consequence, the preconditions for the emergence of BEF relationships change dynamically and so do the relationships themselves.

*Hierarchical nature of trait influence:* Relating functional traits directly to ecosystem functions jumps over several levels of the organismic hierarchy. Traits are related to individual performance, vital rates of populations, community assembly or finally ecosystem functioning [31–34]. For example, maximum assimilation rate is a good predictor of individual growth rates [35] and seed traits also govern population and community-level phenomena [36]. The effects of traits at lower levels can propagate to higher levels, but the importance and direction of trait effects may change along the hierarchy. Unravelling the mechanistic underpinnings of BEF relationships requires tracing trait effects across hierarchical levels; failure to do so may blur rather than elucidate the relationships.

There are currently no experiments or datasets that would allow a comprehensive mechanistic analysis of the interplay of succession, biodiversity and forest biomass dynamics by acknowledging the multi-faceted, dynamic and hierarchical nature of trait influences. This would require a centuries-long biodiversity experiment accompanied by trait and process measurements at the individual, population, community and ecosystem level. Forest inventory data cover longer time scales than current experimental studies [27,37–41]. However, the individual data points only represent snap-shots of unknown successional trajectories, rendering a space-for-time reconstruction impossible. Furthermore, unlike experiments, they are confounded by environmental heterogeneity and are restricted to available gradients and combinations of species richness and identity, and therefore generally lack the spread and orthogonality necessary to disentangle functional diversity and identity effects over time.

Here, we use the trait-calibrated, process-based, dynamic vegetation/ecosystem model LPJ-GUESS [42,43] to gain a mechanistic understanding of the effects of FC on ecosystem productivity by tracing the processes from traits over individual performance, community assembly to stand-level ecosystem state and rate variables. LPJ-GUESS combines the details in energy and matter balances from DGVMs (Dynamic Global Vegetation Models) [44] and the demographic processes of forest dynamics from forest gap models [45]. This way, we may overcome some of the above-mentioned shortcomings, allowing us to study the effect of any FC over any time on a wide range of ecosystem functions [31,46–48]. This approach has also been taken by Morin *et al.* [47], who used a multi-trait model taking into account observed trade-offs in species biology to model forest biomass dynamics over a gradient of species richness.

We here advance their approach by resolving the dynamics hierarchically and tracing all trait effects in a closed framework of a path analysis (see figure 4). This mirrors the ‘traits, states and rates’ (TSR) framework of Purves & Vanderwel [49]. The TSR framework says that traits of individuals together with the current states of the community determine the rate of change of the states (rates). We partition ecosystem processes into a directed graph from functional traits to ecosystem states and rates. The traits are captured in the measures of functional richness, dissimilarity and identity of the community. The states are relevant descriptors of the forest structure, namely leaf area index (LAI), height, biomass and water stress. The rates describe biomass fluxes: growth, mortality, recruitment as well as leaf and root turnover. We then trace the path of biomass change via the rates and states finally to the different facets of FC. This way, we do not need to perform tedious sensitivity analyses and can directly pinpoint specific mechanisms underlying BEF relationships in forests—over long time scales and across the entire hierarchy.

We parametrized the model based on 22 functional traits of 31 temperate deciduous tree species. The trait space was simplified to a single trade-off. This trade-off spanned a gradient from early to late-successional species and thus is especially suited to reveal successional dynamics of BEF relationships. As a heuristic strategy to capture this functional gradient on a species level, we created 16 pseudo-species that were distributed along the trade-off. Using this species pool, we assembled sets of species that represented combinations of functional richness, dissimilarity and identity that were as independent as possible in order to be able to disentangle their respective contributions to the biomass balance of the forest. The modelling approach also allowed us to quantify *biodiversity effects*—as the difference between performance of species mixtures and the null model of abundance weighted monoculture performance—over the entire succession and for all relevant processes.

With this model framework, we expect to see that (i) the three facets of FC affect biomass balance differentially and independently, (ii) the strength and direction of BEF relationships vary over the course of forest succession and (iii) this is due to the varying importance of mediating rates and states at lower hierarchical levels during succession. We present a dynamic and hierarchical framework for analysing BEF relationships that copes with the inherent complexity of natural ecosystems.

## 3. Material and methods

### 3.1 Vegetation model

We used the dynamic vegetation/ecosystem model LPJ-GUESS [43]. LPJ-GUESS has been used in a variety of studies addressing, for example, continental tree species distribution [42], regional vegetation dynamics [50], global water balances [51], continental fire disturbance [52], regional storm damage [53] and regional and global climate change effects on vegetation [54,55], but not yet for BEF studies. LPJ-GUESS and the closely related LPJ have been validated by comparison with field observations in a large number of studies (cf. www.nateko.lu.se/lpj-guess and www.pik-potsdam.de/research/projects/lpjml).

LPJ-GUESS simulates vegetation structure and composition in response to spatial and temporal variation in temperature, precipitation, incoming radiation and soil physical properties. Spatial heterogeneity of forest structure is accounted for by simulating a number of replicate patches (0.1 ha) that all have the same climate and soil type but differ in their disturbance history and stochastic processes such as tree recruitment and mortality. The vegetation in each patch is represented by tree cohorts, where trees of the same age and species are represented by an ‘average’ individual [53].

Physiological processes (e.g. photosynthesis, plant respiration and microbial decomposition) and associated fluxes of carbon and water between soil layers, vegetation and the atmosphere are simulated on a daily time step. Growth and vegetation dynamics are updated by allocating the annually accrued net primary production (NPP) to leaves, sapwood and fine roots according to a set of allometric rules. Growth, sapwood-to-heartwood conversion, litterfall, fine root turnover, recruitment and mortality are all simulated annually [44,50]. We added a storm mortality process following Lagergren *et al.* [53] and a crushing mortality process, where trees are crushed by other falling trees. Both were parametrized based on Holzwarth *et al.* [56]; see the electronic supplementary material, Appendix A for details.

Species are characterized by different static parameters (equivalent to functional traits), such as bioclimatic limits, allometric relationships, tissue C : N ratios and physiological, morphological, phenological and life-history criteria governing dispersal, growth, mortality and competition for light and water (but not nutrients). A complete description of model equations is given in Smith *et al.* [43] and Gerten *et al.* [51]. Here we used the LPJ-GUESS v. 2.1 [54] with changes to disturbances and mortality causes and slight alterations of allometry and recruitment. Details of the changes are documented in the electronic supplementary material, Appendix A.

### 3.2 Functional traits, species pool and pseudo-species

We selected 31 broadleaved woody species that commonly occur in central European forests. The 22 functional traits that we used in this study were derived from our own forest inventory data [56–58] and obtained from the scientific literature and trait databases (via the TRY-database [59]). A complete list of species and traits with respective values and references can be found in the electronic supplementary material, Appendix B.

**Figure 1.**
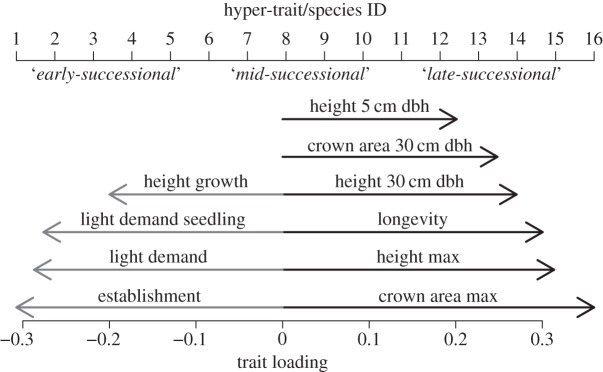
Trait loadings of first PCA axis and species ID along hyper-trait gradient. Trait loadings on first axis (26% of total variation explained; black, positive; grey, negative loading). Just the 10 most influential traits are shown for brevity. For trait details cf. electronic supplementary material, Appendix B. Above the arrows: the hyper-trait axis scaled to a range of 1–16 and the position of the pseudo-species on that axis. The ID of a species equals its hyper-trait value.

To reduce the dimensionality of the trait space, we performed a principal component analysis (PCA). Axis 1 was related to crown area, maximum height, recruitment rate, longevity, light demand and height growth (figure 1). Axis 2 was characterized by the traits height to dbh (diameter at breast height) allometry, storm resistance, wood density, C : N ratio of the wood and drought tolerance. Axis 3 was mainly related to crown area to dbh allometry, specific leaf area (SLA), sapwood conductivity, fire resistance and height growth. The degree of explanation of the first three axes was 26%, 17% and 14%, respectively. We chose to use only the first axis, because it was most strongly related to the key processes in our study system, a temperate forest. The first axis distinguishes early versus late-successional species. Early-successional species are characterized by a small crown, short maximum height, high recruitment rate, short lifespan, high light demand and fast height growth. Late-successional species have opposite traits. We created 16 pseudo-species that we arranged evenly spaced along the empirical trade-off represented by the first axis of the PCA (figure 1). We will refer to this first axis as hyper-trait and the pseudo-species on it as: ‘early-versus late-successional’ and will further speak only of ‘species’ for simplicity. We scaled the values of the hyper-trait to a range of 1–16, so that the each species' trait value equals its position on the hyper-trait axis and its ID. From these values, all measures of FC were calculated.

### 3.3 Experimental design and site

We chose three measures of FC that represent roughly independent facets of the functional trait distribution: potential range (functional richness (F-Ric), measured as the distance between the most extreme species on the hyper-trait axis, taking values from 0 (monospecific stands) to 15), spread (functional dissimilarity (F-Diss), measured as Rao's Q of the hyper-trait) and location (functional identity (F-ID), measured as the community weighted mean of the hyper-trait, CWM, taking values from 1 to 16) [60,61]. F-Ric depends just on the species pool and is thus constant over time, while the other indices depend also on species abundance and thus change over time. F-Ric marks the extremes and thus the potential shift of F-ID and F-Diss.

Biodiversity effects may be mathematically partitioned into selection and complementarity effects by adapting the method of ‘additive partitioning’ [62]. Complementarity effects are linked to all three facets of FC [62,63], selection effects are linked mainly to F-Ric in that the higher F-Ric the higher the probability of including a dominant species [20] and also to F-ID as it is mostly determined by the trait values of dominant species [26,63]. In our analysis, we chose to partition diversity effects over the three facets of FC and performed additive partitioning only for comparison with other studies.

**Figure 2.**
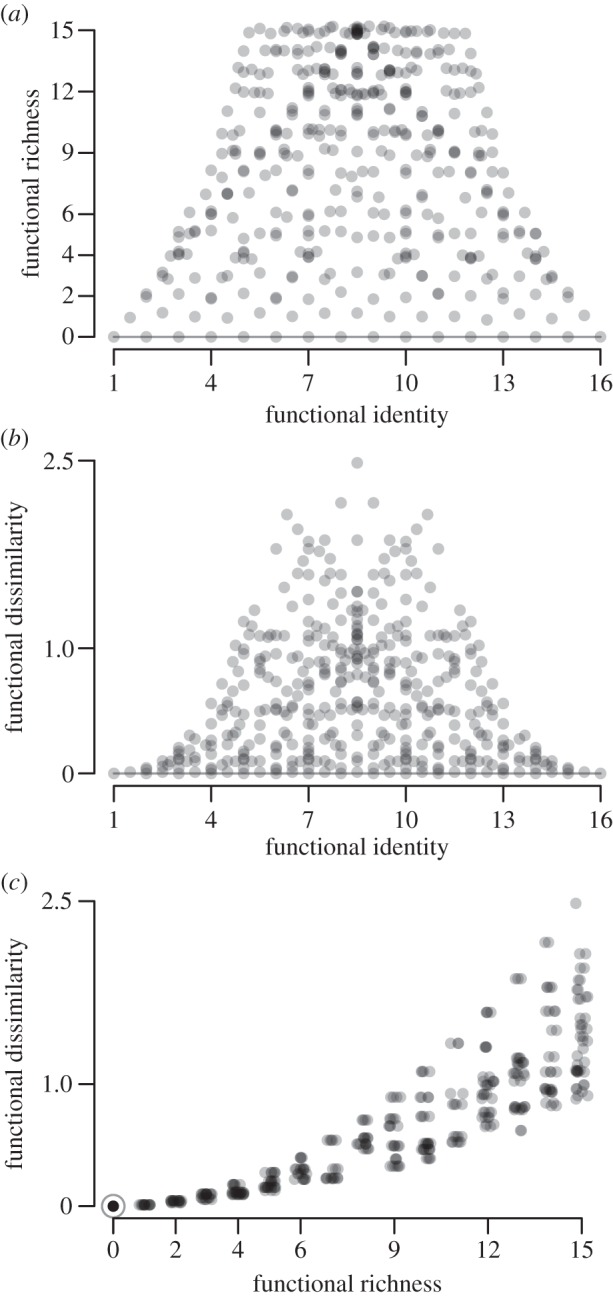
Species pools in the scenarios. Each dot represents one out of 416 scenarios. Functional identity and dissimilarity were calculated with equal abundance for each species. The species pools for the scenarios were chosen as to cover the space spanned by functional richness, dissimilarity and identity as completely as possible. The 16 single-species runs are marked with a surrounding circle or a continuous line, a jitter was added in (*a*,*c*) to make overlaying scenarios visible.

Our study aims call for an orthogonal experimental design, where the different facets of FC (richness, dissimilarity and identity) are as little correlated as possible, so their influences may be separated. We thus designed a set of 400 initial species pools, where the space spanned by F-Ric, initial (that is, each species receives the same weight) F-Diss and initial F-ID was covered as completely as possible (figure 2 and electronic supplementary material, table S1 and figure S1 in Appendix D). These species pools could be regarded as trait-based extinction scenarios, i.e. diversity gradients being created by removing species according to a rank-order of the hyper-trait value. Additionally, 16 single-species runs were conducted to create a null model as reference (see below). We chose species richness to be 1, 2, 3, 4, 6, 8, 12, 15 or 16 species. We simulated ecosystem dynamics for each of the 416 species pools for 500 years starting from bare ground and over 200 patches each (20 ha in total).

All model experiments were run on a single site. As locality we chose coordinates from a nature-reserve in Central Germany, the Hainich National Park (51^°^N, 10.5^°^E) from which we have plenty of inventory data (used to derive species traits and for preliminary validation purposes not shown here). Environmental drivers (climate, storm and fire regime) were the same for every run.

### 3.4 Analysis of model experiments

**Figure 3.**
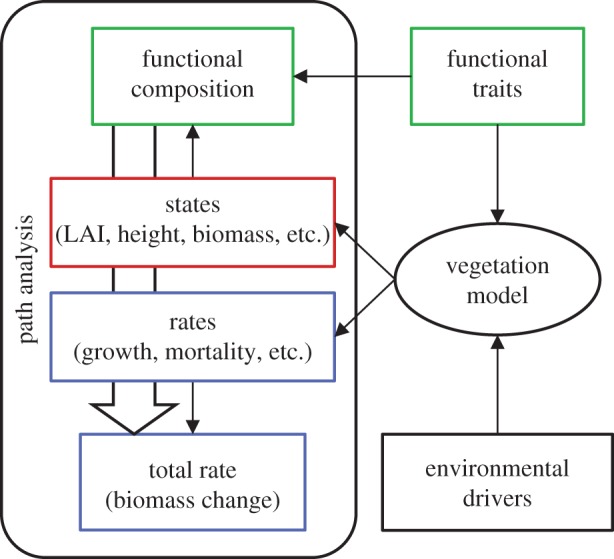
Analysis scheme. The epistemic rationale of the study is to simultaneously generate ecosystem states and rates as well as functional composition using a vegetation model, which is fed by empirical functional traits, and then to trace effects of functional composition on ecosystem functions (states and rates) by means of path analysis. The rates (growth, recruitment, leaf and root turnover, mortality) sum up to the total rate of vegetation biomass change.

We chose annual biomass change, ABC (kg C m^−2^ yr^−1^), of the vegetation as a measure of ecosystem functioning, as it integrates over all carbon fluxes in and out of the vegetation and thus is a good monitor for this important component of the ecosystem carbon balance. We traced effects of FC to ecosystem functioning using the vegetation model as a surrogate for nature (figure 3). The vegetation model was fed by empirical functional trait data and information on environmental drivers. It rendered dynamic output of ecosystem states and rates as well as information on current FC. Mirroring this, we used statistical path analysis to trace from FC to ecosystem functioning via intermediate states and rates [64,65]. The ‘traits, states and rates’ scheme [49] served as a blueprint for the path analysis, the traits were represented in the analysis via indices of FC and the explanatory and target variables (states and rates) came from the vegetation model output.

**Figure 4.**
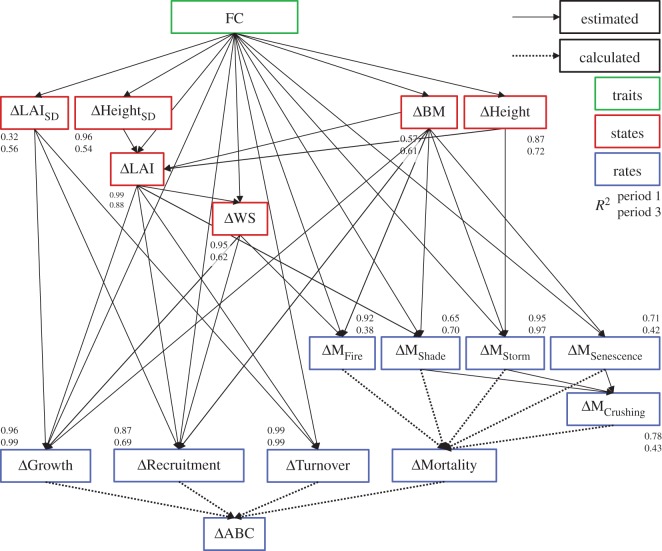
Path model used to analyse the effect paths of FC on the target variable ΔABC (biodiversity effect in ABC) via intermediate states and rates. All states and rates represent the biodiversity effect in them (difference between observation and null model expectation), denoted by a leading Δ. Solid lines were estimated with linear regressions as part of the path modelling. Dotted lines were calculated, such that ΔABC was the sum of ΔGrowth, ΔRecruitment, ΔTurnover (leaf and roots) and ΔMortality and ΔMortality was the sum of five mortality processes. The model fits (*R*^2^) are placed directly adjacent to the estimated variables with the model fit for period 1 above and period 3 below. FC comprises functional richness, functional dissimilarity and functional identity. States are BM, biomass (kg C m^−2^); Height, tree height (m); LAI, leaf area index (m^2^/m^2^); LAI_SD_, the standard deviation of LAI across patches and years in a time period, Height_SD_ the standard deviation of Height in a patch and WS, a metric between 0 and 1 indicating water stress. Rates (kg C m^−2^ yr^−1^): ABC, annual biomass change; Growth, growth of existing trees; Recruitment, growth of new regeneration; Turnover, turnover of roots and leaves; Mortality, mortality of living trees; M, mortality due to five processes: Shade, Senescence, Storm, Fire and Crushing. We analysed the biodiversity effect on states and rates, indicated by a leading Δ. Where applicable, we used averages weighted by tree and/or patch biomass. This figure omits some intermediate rates (such as e.g. NPP) that are not discussed in this paper, but see electronic supplementary material, Figure C1 in Appendix C.

We focused on states and rates that, based on formulations in the vegetation model or on previous knowledge, should have a major impact on ABC. Biomass is added to the vegetation by tree growth and recruitment of new trees. Biomass is lost from the vegetation through leaf and root turnover and tree mortality. Likewise, we selected states that either resemble important states in the vegetation model (water stress) or translate into measurable quantities of forest structure, such as LAI, height, biomass and variability thereof (LAI_SD_, Height_SD_ (SD denotes standard deviation)). The variability was included to capture horizontal (LAI) and vertical (height) heterogeneity that in both cases may affect the effectiveness of light capture [47]. In the case of LAI_SD_, the effect is decreased light interception with increased variability due to the nonlinearity of light interception (Beer–Lambert Law), while for Height_SD_ it is vice versa due to an extended vertical space filling. The path model links all traits (as facets of FC), rates and states via either linear regressions or mathematical equations (figure 4). All equations are given in the electronic supplementary material, Appendix C.

In the analysis, we focus on tracing the biodiversity effect in ABC (ΔABC) to the different facets of FC. *Biodiversity effects* (Δ) describe the difference between observed species performance when growing together in mixtures and expectations based on monoculture performances. The expectations were derived from a null model [62]. This uses the monospecific variable (e.g. biomass) of the species as a reference and their relative abundance in terms of biomass in the mixture as the multiplier

$$X_{\mathrm{null},t}=\sum_{i=1}^{n}X_{\mathrm{mono},i,t}\cdot \frac{{\mathrm{BM}}_{\mathrm{mix},i,t}}{{\mathrm{BM}}_{\mathrm{mix},t}},$$

with X_null_ the expectation of a variable X in the null model, X_mono,*i*_ the variable X of species *i* in the monospecific stand, BM_mix,*i*_ the biomass of species *i* in the mixture, BM_mix_ the total biomass (BM) in the mixture, all at the time *t*, and *n* the number of species in the mixture. We calculated the biodiversity effect in each variable as the difference of the mixture simulation (observation) to the null model expectation

$$\Delta X_{t}=X_{\mathrm{mix},t}-X_{\mathrm{null},t}.$$

The Δ-notation thus represents the biodiversity effect. We calculated the biodiversity effect not only for our target variable (ABC) but also for all intermediate states and rates (figure 4).

We applied path analysis as a tool of structural equation modelling (SEM) [64,65] to trace effect paths from FC to our ecosystem functioning target variable (ΔABC). Path analysis is apt to deal with the intrinsic process hierarchy and nestedness of variables (states and rates influencing each other) and allows aggregating individual pathways along states or rates of interest.

Path analysis and other analyses and plotting were performed with the software WinBUGS v. 1.4 [66], R v. 3.1.2 [67] and the packages R2WinBUGS [68] and FD [60]. The code for path modelling and path analysis can be found in the electronic supplementary material, Appendix E.

To analyse the model output of ecosystem states and rates and their relationship to FC, the scenario length of 500 years was split into four time periods (1–20, 21–50, 51–100, 101–500 years) and annual model output was averaged over the years in these intervals. The intervals were chosen because ΔABC changed its sign for most model runs around the chosen limits, and as to get a finer resolution in the initial periods, where most of the successional dynamics occur.

For the interpretation of the results of the path analysis, we aggregated its output at different levels. First, we aggregated over all paths from the FC metrics to ΔABC (‘complete aggregation’). Second, we aggregated along the four main rates that directly add up to ΔABC: ΔGrowth, ΔRecruitment, ΔTurnover and ΔMortality (‘aggregation via rates’). Third, we aggregated along the six ecosystem states: ΔLAI, ΔLAI_SD_, ΔHeight, ΔHeight_SD_, ΔBM and ΔWS (‘aggregation via states’). And fourth, we aggregated along four different key mortality processes: Shading, Senescence, Storm and Fire (‘aggregation via mortality processes’). For convenience and better comparison, all variables were standardized to a mean of 0 and a standard deviation of 1. The aggregated paths are commonly quantified by their standardized path coefficients (SPC). In the text, we refer to |SPC|<0.2 as weak, ≥0.2 and <0.5 as intermediate (or without any modifier) and ≥0.5 as strong effects [69], and report only |SPC|≥0.1 and which were significant at a credible level of 95%. We also calculated model fits (*R*^2^) for all the regressions in the path model.

## 4. Results

### 4.1 Successional dynamics of biomass and functional composition

**Figure 5.**
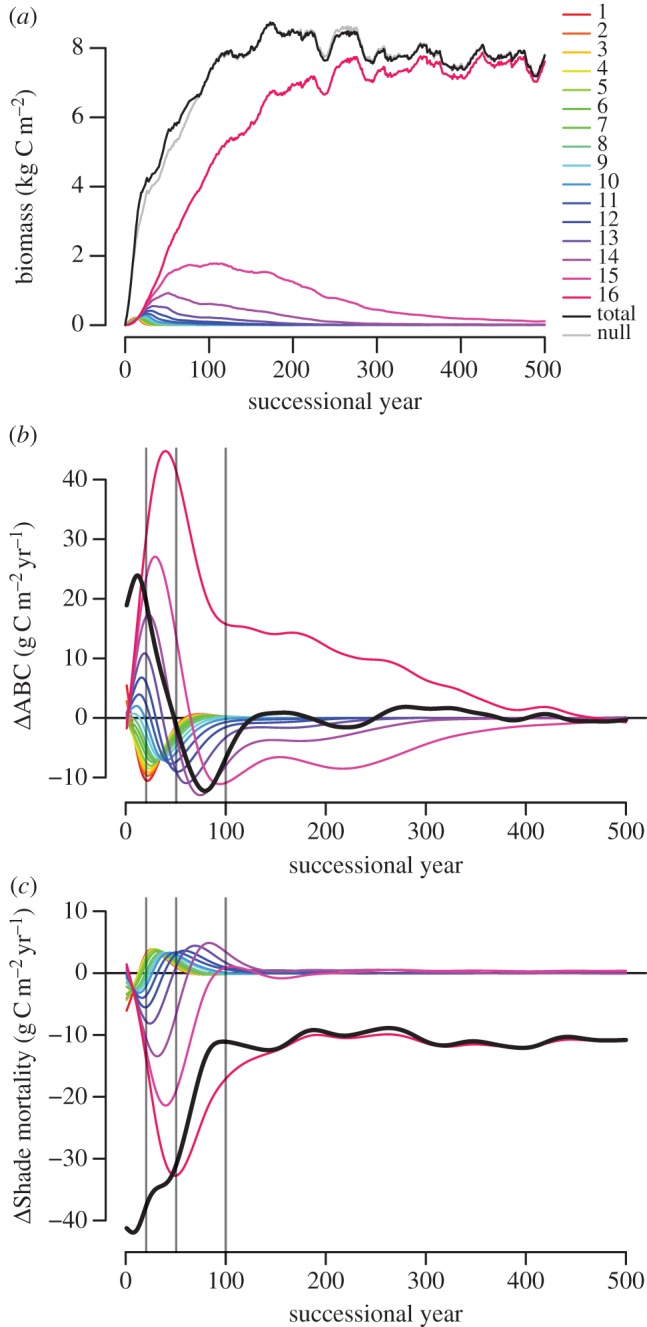
Successional development of the stand with all 16 species. Coloured lines each represent a species with their colour indicating their ID. In (*b*–*c*) lines were smoothed to make trends clearer. Vertical lines separate the four different time periods. A leading Δ denotes the biodiversity effect in the following variable (difference between observation and null model expectation). (*a*) Development of individual and total biomass (kg C m^−2^) over time. Black line: total biomass (BM) observation, grey line: total BM expected according to null model. (*b*) Development of individual and total ΔABC (g C m^−2^ yr^−1^) over time. (*c*) Development of individual and total ΔShade mortality (g C m^−2^ yr^−1^) over time.

In the run with all 16 species (*full run*), the individual species biomass (BM) peaked in the first 10–120 years analogous to their arrangement along the hyper-trait for species 1–15, declining thereafter (figure 5*a*). The most late-successional species 16 surpassed all other species by the year 25, by the year 100 it represented *ca* 60% of the total BM, species 15 *ca* 25% and species 14 *ca* 10%. After 300 years, species 16 was predominant, leaving only a declining residue of species 15. Total BM increased very steeply in the beginning, then declined slightly and saturated at the end of the scenario. The corresponding null model exhibited a lower BM in the first 100 years, but a similar BM afterwards.

A positive biodiversity effect in ABC (ΔABC; Δ-notation refers to biodiversity effect in the following variable) was observed in the first 50 years. Then the effect was negative until about year 120 and from then on close to zero (figure 5*b*). Partitioned into species, early-successionals showed a very early positive contribution to total ΔABC that quickly turned negative. This negative contribution coincided with a strong positive contribution of mid- and late-successionals, which later turned negative, too. Only species 16 maintained a positive contribution, which disappeared at the end of the simulation. Significant parts of these contributions were due to effects via a reduction of shade mortality: while early-successional species tended to suffer from increased mortality, this was more than compensated for by reduced mortality in late-successionals (figure 5*c*).

**Figure 6.**
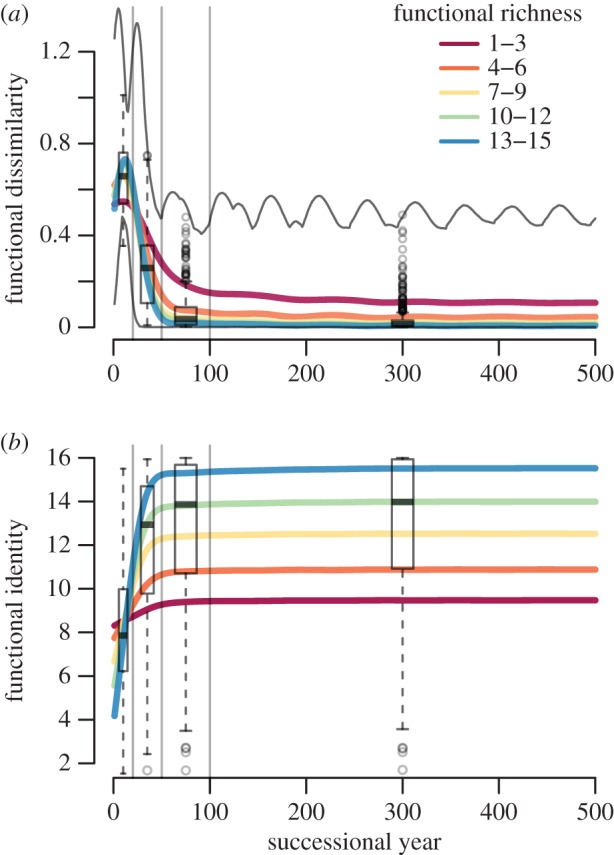
Development of functional composition during forest succession. Vertical lines separate the four different time periods. Functional dissimilarity and identity were averaged annually over the scenarios grouped by functional richness (range of the hyper-trait, which remains constant), as indicated by the colours. (*a*) Functional dissimilarity (Rao's Q). Grey lines mark lower and upper extremes of Rao's Q. (*b*) Functional identity (CWM of the hyper-trait). Boxplots indicate the distribution in the time periods (median as bold line, hinges as interquartile ranges (IQR) and whiskers extend from there to the extremes or 1.5 times the IQR, whichever is shorter, beyond that single runs as points).

Across *all mixture scenarios*, functional dissimilarity (F-Diss) increased in the first 5–30 years, at which point late-successional species became about equally abundant as early-successionals in most scenarios (figure 6*a*). It then decreased to low levels for most scenarios, approaching nearly monospecific stands. Only in mixtures restricted to early-successional species, F-Diss oscillated on intermediate levels. Functional identity always changed to the position of the (most) late-successional species in the species pool in the first 100 years with lower starting values and a steeper rise for functionally richer communities (figure 6*b*). In this way, a high functional richness (F-Ric) allowed for the development of late-successional monocultures dominated by species with higher hyper-trait values. This can be considered a long-term successional selection effect.

**Figure 7.**
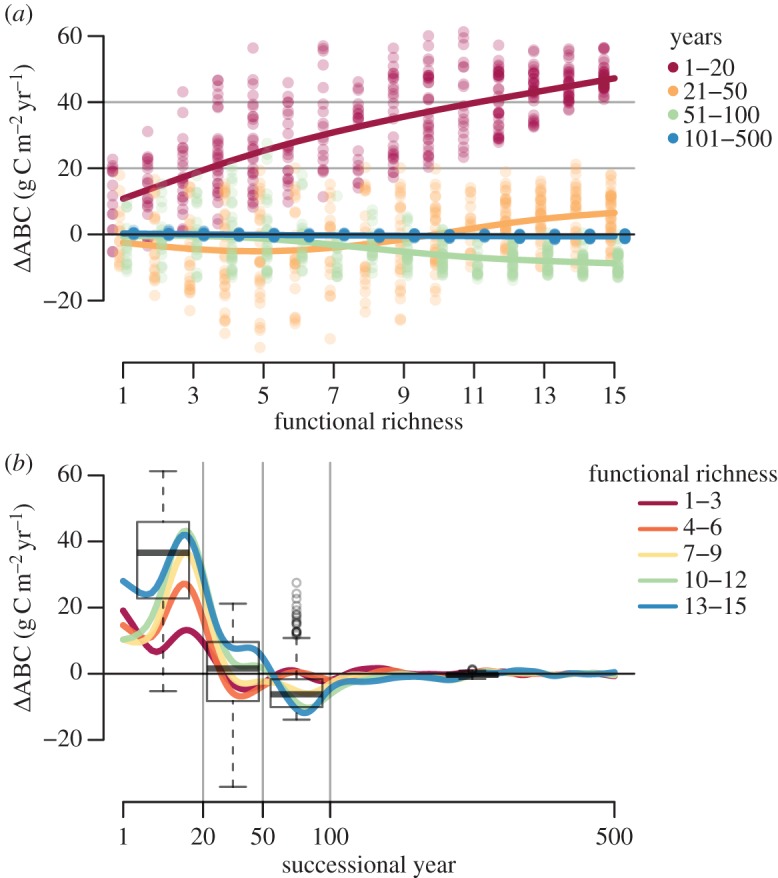
Biodiversity effect in ABC (ΔABC) over functional richness and time. (*a*) Over functional richness (range of the hyper-trait), with a small shift to enhance visibility. Each dot represents the average from a scenario run over the time period. Time periods are shown in different colours. Dots are overlaid by smoothed splines to guide the eye. (*b*) Over time, smoothed splines show annual averages in five groups of functional richness, as indicated by the colours. Boxplots indicate the distribution in the time periods (median as bold line, hinges as interquartile ranges (IQR) and whiskers extend from there to the extremes or 1.5 times the IQR, whichever is shorter, beyond that single runs as points). Vertical lines separate the four different time periods.

In period 1 (1–20 years), ΔABC was nearly always positive across the mixtures and increased with functional richness (F-Ric) of the community and did not reach saturation at the maximum F-Ric (figure 7*a*). In period 3 (51–100 years), ΔABC was negative on average and tended to increase in magnitude with F-Ric. In the periods 2 (21–50 years) and 4 (101–500 years), ΔABC was close to zero on average. However, in period 2, there was a higher variability, indicating a varying point in time, where the shift from positive to negative effects occurred, and in period 4 there was virtually no variability (figure 7*b*).

### 4.2 Effect pathways of functional composition on biomass change

We only analysed time periods where there was a significant deviation from the null model expectation and thus potentially a significant impact of FC on biodiversity effects in ABC (ΔABC). The time periods had to meet two criteria: (i) the median of the relative biodiversity effect (ΔABC/ABC_null_) was more than 10% or less than −10% across the simulations and (ii) the first and third quartiles of the relative biodiversity effects had the same sign (either negative or positive). This was the case in time periods 1 (1–20 years) and 3 (51–100 years) with the median (and quartiles) being +24% (14%, 32%) and −16% (−24%, −8%), respectively (figure 7*b* and electronic supplementary material, table S2 in Appendix D).

**Figure 8.**
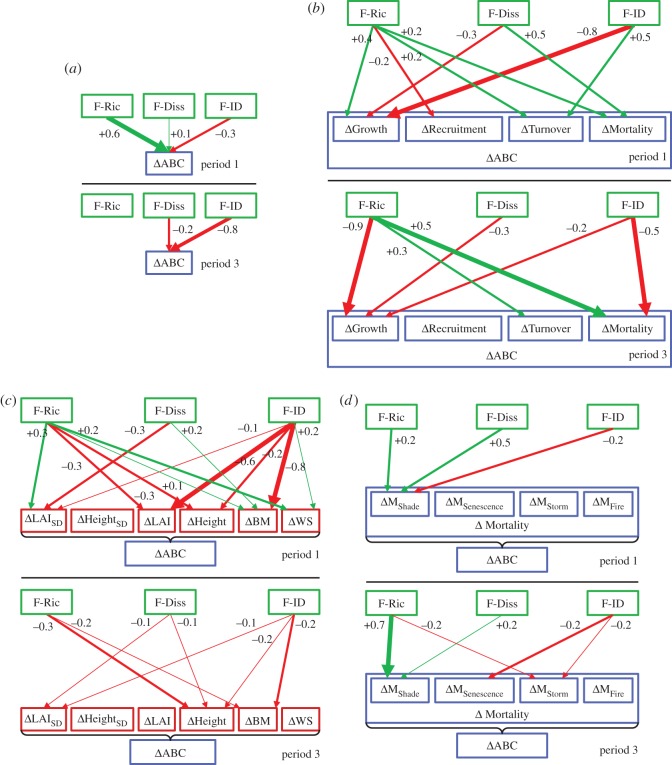
Aggregated results from path models for periods 1 (top) and 3 (below). With four versions of aggregated effects (SPC) of FC metrics on biodiversity effects in ABC (ΔABC): (*a*) ‘complete’, (*b*) ‘via rates’, (*c*) ‘via states’ and (*d*) ‘via mortality processes’. A leading Δ denotes the biodiversity effect in the following variable (difference between observation and null model expectation), which applies here to every variable except for FC. Arrow colours reflect the sign (green, positive; red, negative) and boldness the strength of the SPC with line widths 1–3 corresponding to |SPC|≥0.1 and <0.2 (thin), ≥0.2 and <0.5 (medium), ≥0.5 (bold); only significant paths and paths with |SPC|≥0.1 are shown. The SPC estimates are placed directly adjacent to the arrows. Box colours: green, FC metrics based on functional traits; red, states; blue, rates. In (*b*), the effects via the rates sum up to the total effects on ΔABC, indicated by the surrounding frame, in (*c*) the effects via the states do not. In (*d*), ΔM_Shade_, ΔM_Senescence_, ΔM_Storm_ include their indirect effect via ΔM_Crushing_, such that all four processes sum up to the total ΔMortality, indicated by the surrounding frame. F-Ric, functional richness; F-Diss, functional dissimilarity; F-ID, functional identity; ABC, annual biomass change; LAI, leaf area index; BM, biomass; WS, water stress; M, Mortality.

The aggregated path model results (‘complete aggregation’, ‘via rates’, ‘via states’ and ‘via mortality rates’) for both time periods including the estimates of the aggregated SPC are displayed in figure 8. The model fits can be found in figure 4 and all individual SPC in the electronic supplementary material, table S3 in Appendix D.

#### 4.2.1 Complete aggregation (overall effects)

In period 1, each facet of FC had a significant total effect on ΔABC. Increased functional richness (F-Ric) of a community had a strong positive effect on ΔABC (figure 8*a*1). Increased functional dissimilarity (F-Diss) had a weak positive effect and functional identity (F-ID) towards late-successional species had an intermediate negative effect. In period 3, these effects became more negative. F-Ric lost its positive effect, having no overall effect on ΔABC, the effect of increased F-Diss turned from positive to negative, and the negative effect of late-successional identity became stronger (figure 8*a*3).

#### 4.2.2 Aggregation via rates

In period 1, communities with higher F-Ric showed increased ΔGrowth and reduced biomass loss due to ΔTurnover and ΔMortality, resulting in a strong positive overall effect on ΔABC (figure 8*b*1). Higher F-Diss led to reduced ΔMortality and thus strongly increased ΔABC but at the same time also to lower ΔGrowth and decreased ΔABC. Both effects partly neutralized each other, resulting in only a weakly positive overall effect. Communities with an F-ID towards late-successional identity showed a strong reduction of ΔGrowth but also a strong reduction of ΔTurnover. The overall effect on ΔABC was intermediately negative. In period 3, functionally richer communities showed strongly decreased ΔGrowth, which was the opposite of period 1, but again also reduced ΔTurnover and ΔMortality (figure 8*b*3). The two opposing effects cancelled each other out and F-Ric had no overall effect on ΔABC. The negative effect of higher F-Diss on ΔABC was entirely due to reduced ΔGrowth. As in period 1, higher values of F-ID (late-successional identity) led to a reduction of ΔGrowth and additionally to increased ΔMortality, adding up to a strong negative overall effect. Effects via ΔRecruitment did not play any significant role in either period.

#### 4.2.3 Aggregation via states

The biodiversity effects in ABC were mediated only partly along indirect paths via states (stand structure and water stress) but also directly via rates (cf. figure 4), such that the effect strengths do not sum up to the totals here. In period 1, stands with a functionally richer community (higher F-Ric) showed a more spatial as well as temporal stability of leaf cover (lower ΔLAI_SD_) and less water stress (lower ΔWS), which both led to increased ΔABC (via increased ΔGrowth) (figure 8*c*1). However, those stands tended to also be higher (ΔHeight) and have a lower ΔLAI, both decreasing ΔABC via decreased ΔGrowth. Higher F-Diss weakly increased ΔABC via an increase in stand biomass (ΔBM) (interrelated with its positive effect on ΔABC, which creates a higher biomass) but also decreased it via an increase in ΔLAI_SD_. Communities with a more late-successional identity (higher F-ID) showed reduced water stress, which weakly increased ΔABC. However, they also had lower leaf cover, lower biomass and were higher (ΔLAI, ΔBM, ΔHeight), which had a negative effect on ΔABC. In period 3, the biodiversity effects in ABC via the states were much weaker than in period 1 (figure 8*c*3). Contrary to period 1, higher F-Ric led to reduced ΔABC via an increase of ΔHeight. A more late-successional identity led to reduced ΔABC via increased ΔHeight and ΔBM. However, direct effects via the rates seemed to be more important for ΔABC as functional richness had no overall effect on ΔABC while functional identity had a strong negative effect.

#### 4.2.4 Aggregation via mortality processes

In period 1, the influences of FC on ΔABC via mortality were only due to shade-related mortality (ΔM_Shade_) (figure 8*d*1). Higher F-Ric and F-Diss reduced ΔM_Shade_, thus increasing ΔABC. The opposite was true for late-successional identity. In period 3, the picture was more complex. Again, higher F-Ric and F-Diss strongly reduced ΔM_Shade_ (figure 8*d*3). A more late-successional identity had a negative effect on ΔABC due to increased senescence-related mortality (ΔM_Senescence_). In mixtures without late-successionals, the mid-successionals tended to be younger than in the respective monocultures. This is because in period 2 (not shown) a greater proportion of them died, and so subsequent regeneration led to younger trees in period 3, which experienced lower ΔM_Senescence_. Mortality due to storms (ΔM_Storm_) was also higher in communities with higher functional richness and identity, because of an overall increase in stand height and height variability compared with null model expectations. Fire played hardly any role in either period.

Complementarity effects contributed on average 84% of the total biodiversity effect in period 1 and 73% in period 3. The remainder was thus due to selection effects, whose relative contribution increased over time, saturating at about one-third after around 200 years (cf. electronic supplementary material, figures S2, S3 and table S4 in Appendix D). The selection effect in ABC in period 1 was mostly attributable to F-Ric and not to F-Diss or F-ID (electronic supplementary material, figures S4-I and S5 in Appendix D).

## 5. Discussion

The present study uses a mechanistic model to shed light on the influence of biodiversity—here approximated as functional composition—on the integrative ecosystem function ‘*annual biomass change*’ (ABC) in forest ecosystems. Our approach embraces complexity: it traces trait influence via different facets of functional composition (richness, dissimilarity, identity); it unfolds the time dimension by monitoring trait control of BEF relationships over successional time scales; and finally it resolves the natural process hierarchy into important precursory rates and intermediate states, which allows us to unravel the hierarchical nature of trait control. In the following, we highlight the most important findings and discuss their implications. For readability, we henceforth refer to the biodiversity effects in the variables without the leading Δ.

*Facets of functional composition:* The three metrics capturing important facets of functional composition, namely functional richness (F-Ric), functional dissimilarity (F-Diss) and functional identity (F-ID) (which were not correlated in period 1 and weakly in period 3, cf. electronic supplementary material, figure S6 in Appendix D) all had different magnitudes, pathways and temporal patterns in influencing biomass trajectories. During early succession (1–20 years, period 1), higher functional dissimilarity enhanced resource conservation through reduced shade mortality, while resource acquisition was boosted in species mixtures with a stronger early-successional identity (lower F-ID) through strongly increased growth. Functional richness positively affected both resource conservation and acquisition. During the mid-succession (51–100 years, period 3), stands with higher functional richness again displayed more resource conservation (i.e. decreased turnover and mortality). Resource acquisition (growth) was negatively affected by functional richness and dissimilarity as well as a more late-successional functional identity. This can be explained by a reduced growth of early- and mid-successionals under the canopy of the then dominant late-successional species. The overall effect of functional richness on ABC was not significant. These results contradict our expectation that resource acquisition (growth, recruitment) is more important than resource conservation (mortality, turnover) during early and less important during later successional stages. Rather, both resource acquisition and conservation are equally important for the biomass balance at all successional stages.

From a naive perspective, this just highlights the importance of partitioning functional biodiversity effects into various facets: identity, richness and dissimilarity (or alternatives such as, for example, functional divergence) effects [10,24]. Revealing these links was made possible by unfolding the individual pathways and by sub-dividing the time dimension. Neglecting either of these steps by not zooming into the process hierarchy and disentangling the aggregation of rates or by analysing just one point in time would have led astray. In our case, we would have misinterpreted the relationships, e.g. by taking the non-significant overall effect of functional richness in period 3 as ‘no influence’ at all, or by generalizing the negative effect of functional dissimilarity in period 3 over time and thus overlooking its positive effect in period 1. We will thus also take a closer look at both dimensions: successional dynamics and mediator rates and states.

*Change of effects over successional time:* The change of effect sizes and pathways over successional time emphasizes that biodiversity may have strongly varying influences and that these influences may be mediated by different pathways depending on the successional stage [70]. Here, we restate the examples of the previous paragraph from a temporal perspective. Higher F-Ric was associated with increased growth during early succession (1–20 years, period 1) but strongly decreased growth during mid-succession (51–100 years, period 3), while the negative impact on mortality and turnover remained over time. To the contrary, the negative influence of higher F-Diss on growth was about the same in both periods, but the strong positive effect via reduced shade mortality disappeared in period 3. Lastly, the strong negative effect of a more late-successional F-ID on growth in period 1 diminished in period 3. This indicates that the contribution of the fast growing early-successionals is most important during early succession (cf. ‘shifting trait hypothesis’ *sensu* Wirth & Lichstein [29]). In period 3, a strong negative effect of late-successional identity via increased storm-related mortality appeared. This was due to higher stand heights and more exposed crowns of the late-successionals in mixed stands, which increased the susceptibility to storm damage [53]. As a consequence of these dynamic shifts, biodiversity effects were only positive in the early-successional period and then turned negative during the transitional stage (21–50 years) for most species combinations. It should thus be considered in which successional period we look at biodiversity effects on ecosystem properties. This may be especially important for the design and analysis of forest BEF experiments.

*Importance of mediator rates and states:* We looked at the complete biomass balance, not just growth of established trees, by including new recruitment, annual tissue turnover (leaves and roots) and mortality. While growth was clearly an important pathway via which biodiversity effects were mediated, we also found mortality and turnover to be important mediator rates in different successional stages. In both periods, higher functional richness and dissimilarity led to significantly reduced shade-related mortality. This suggests that biodiversity may positively affect community biomass by both the improved acquisition of resource and the enhanced conservation of what is there. This was achieved due to (i) improved vertical assembly, where shade-tolerant late-successionals grew under the shading canopy of light-demanding early-successionals; due to (ii) improved species assembly, where a proportion of light-demanding early-successionals were replaced by shade-tolerant late-successionals that grew slower in height but survived, while the early-successionals received more light because of lower densities; and finally due to (iii) reduced tissue turnover of the late-successionals. Mortality has rarely been studied as a process in BEF research and, where studied, was not found to play any role in mediating biodiversity effects at the community level. Species identity but not diversity played a significant role in mortality for a tropical tree plantation [71], mortality related to an ice storm in a subtropical forest [11] and overall mortality in US forests [37]. By contrast, in the modelling study of Morin *et al.* [47], mortality increased with species richness as a result of increased shading in more species-rich forests. To recognize mortality as an important mediator of BEF relationships, it may be necessary to disentangle different mortality processes (e.g. shade versus others) and successional periods as well as facets of functional composition. Further observational and experimental research is urgently needed as mortality is, by its sporadic nature, a more elusive phenomenon than growth.

Biodiversity effects were partly mediated via components of stand structure. These represent metrics of space filling [72,73] and are thus related to complementary resource use. As examples from period 1 (1–20 years), higher functional richness led to increased growth, partly because of reduced water stress (cf. [74]) and reduced temporal and spatial variability of leaf cover (LAI_SD_) (cf. [75]). Higher variability of leaf cover at a given mean leads to lower rates of light interception because of the nonlinear light interception curve. This indicates that temporally stable as well as spatially homogeneous LAI ensures better light capture and thus more growth than a more heterogeneous crown cover, due to e.g. large gaps. Higher functional dissimilarity led to reduced growth, mainly via an increase in LAI_SD_, while early-successional functional identity was associated with strongly increased growth, mainly via an increase in LAI. LAI and water stress being important mediators for tree growth is supported by long established evidence [73,76,77]. LAI summarizes the above-ground organization of leaves and the potential for light capture, whereas water stress is related to rooting patterns and depths, and hence captures below-ground competition for water and nutrients [78]. Consistent with Morin *et al.* [47], we found that not only the mean but also the variability of LAI was a relevant predictor of biomass change. This corroborates the assertion that the variability of structural components needs to be taken into account, especially when the underlying processes are nonlinear, such as light capture.

The structural components of the forest are not independent from each other, e.g. height and standing biomass influence LAI through allometries. In addition, they are also directly or indirectly linked to biomass fluxes. For example, biomass is directly related to growth and mortality, and average tree height has an influence on the susceptibility of trees to storm-related mortality [53]. During early succession, we clearly identified the crucial structural states for the overall biomass balance (biomass, height, LAI, variability of LAI, and to a lesser extent water stress). However, at the later successional period, the direct importance of structural states for biomass balance diminished.

Recent BEF studies, while already using sophisticated analysis methods such as SEM, have still made strong simplifications. Either they assumed a direct effect of functional composition on highly aggregated ecosystem functions such as productivity (e.g. [38,39,79]) or, when indirect effects were included, they were mediated by just one single step, such as tree density [79] or basal area [38,39].

Here we have demonstrated that the effects of one facet of functional composition at lower hierarchical levels can counter or even cancel each other at a higher level. A perspective that ignores the hierarchy in functions and pathways may miss ecologically important mechanisms. Skipping levels in the process hierarchy may just lead to coarser mechanistic resolutions, as long as no process on the same hierarchical level is omitted (e.g. to asses vegetation biomass change, not only growth but also mortality needs to be accounted for). However, when processes on the same level are missed, this may lead to incomplete or flawed predictions of BEF relationships. This would be the case, for example, when taking growth as the sole component of vegetation biomass change and ignoring mortality or when certain rare but influential mortality events, such as storms or fires, were not sampled [80]. Taking a modeller's viewpoint by pinpointing possible mediating structures, by hypothesizing causal networks [81] and by accounting for all major contributions to mass balances [82] could thus improve real-world study design and analysis. In the context of real-world systems, other processes increase complexity, such as, for example, diseases, insect damages, browsing, nutrient uptake or small-scale variation in climate, topography and edaphic conditions. Thus, possibly more states need to be observed to form a comprehensive picture of biomass fluxes and biodiversity effect pathways.

### 5.1 Validation

In all model experiments, functional dissimilarity (F-Diss) decreased over time as early-successional species became rare and late-successional species took over, thus changing also functional identity (F-ID) towards higher values [29,46]. These are expected changes in forests with infrequent disturbances and a strong functional separation of species into early- and late-successional species (as we imposed with the species being arranged along a hyper-trait).

As an external reference, we compared the absolute values of annual biomass change (ABC) over the whole simulated succession (500 years) with observed and simulated data from Wirth & Lichstein [29] as well as biomass trajectories with simulated data from Kinzig & Pacala [46] (electronic supplementary material, figures S7 and S8 in Appendix D). The magnitude as well as the pattern of ABC development over time was similar, except that our model exhibited lower values in the late transitional stage (101–200 years), which might be due to the restricted species pools we used. The biomass trajectories of early-, mid- and late-successional species were again very similar.

Morin *et al.* [47] found a positive relationship of functional dispersion with the relative complementarity effect and none with the selection effect in a similar model experiment. Our results confirmed this relationship with F-Diss (electronic supplementary material, figure S4 in Appendix D, functional dispersion tends to be very tightly correlated to F-Diss [60]). Our finding that functional richness induces a selection effect is consistent with the view of Díaz & Cabido [20], however we could not confirm any relationship of the selection effect with F-ID, as e.g. Mokany *et al.* [26], Roscher *et al.* [63] and Ruíz-Benito *et al.* [27] suggested. Rather, our results do not support the notion of F-ID representing the trait-analogue to selection effects (electronic supplementary material, figure S5 in Appendix D). Many previous studies related biodiversity effects to species richness rather than to measures of functional diversity. Likewise, we also found an increase of the biodiversity effect in ABC with increasing species richness and saturation was reached with a richness of six species (electronic supplementary material, figure S2 in Appendix D), which is consistent with the findings of Zhang *et al.* [41] (a global meta-analysis). The magnitude of the relative biodiversity effect in ABC that we found in period 1 ranged from −4 to 44% and was 24% on average. That is comparable with empirical values of 24% from a global meta-analysis [41] and 25–50% from classical forest management experiments [83].

### 5.2 Methodology

The construction of pseudo-species allowed for a strictly functional design of the model experiments without the peculiarities, and thus imbalances, of real species that would otherwise confound the controlling regime of functional composition. The effects of functional composition on ecosystem functions were already strongly expressed with just one functional axis describing species differences. If more dimensions of species differences were taken into account, more possibilities for complementarity between the species could arise. In our case, these could be related to traits that were not or only weakly reflected in the hyper-trait (e.g. drought tolerance, wood density, resistance to fire and storm, leaf phenology and longevity, specific leaf area). Accordingly, we assume that our estimates of biodiversity effects are still conservative.

With our approach, we presented a fusion of trait-based biodiversity research with vegetation modelling, where we adapted the ‘traits, states and rates’ scheme from Purves & Vanderwel [49]. This way, we reach a comprehensiveness that is hardly achievable in observational studies. With this analysis method, we were able to trace the paths from rates and states of the simulated forest to facets of functional composition by means of path analysis. We assume that the vegetation model captures the relevant ecological processes for our research question in a realistic fashion. Our statistical model that mirrors the vegetation model may then be used to unravel effect pathways that are hard to observe in nature (e.g. the effect of functional identity via LAI and growth on biomass change). However, the primary epistemic restriction of this approach lies in the limited representation of processes in the vegetation model and knowledge of non-trait model parameters. Other limitations to simply transfer our findings to real-world ecosystems ensue from the uncertainty in plant functional traits and lacking representation of their intra-specific variability, plasticity over time and interdependence [48]. Adopting the phrase of Morin *et al.* [47], we conclude that ‘our findings provide a significant step towards disentangling the underlying mechanisms of the biodiversity effect on forest productivity’.

Path models that are used in empirical studies are generally simpler (e.g. [38]), as their true structure is unknown and the proposed structure represents a hypothesis based on prior knowledge to be tested with empirical data [65]. However, here we have a sound understanding of the model structure, as we derived it from the vegetation model code. Path analysis, as an out-of-the-box tool, is limited to linear relationships. However, with our method of path modelling using WinBUGS (a Monte–Carlo simulation method) this limitation does not apply. Visualization of the data revealed that all relationships were close to linear with one exception so we used linear regressions throughout and do not believe that this simplification affected the interpretation of the data. However, we caution against automatically assuming linearity in data relationships, which many statistical methods require.

## 6. Conclusion

The structure of cause–effect pathways we found may be conducive to generating new hypotheses and informing the design and analysis of BEF experiments and observational studies. While many studies are already based on extensive trait information of their species pools and distinguish facets of functional composition in their analysis, the other two dimensions of complexity (dynamic and hierarchical trait influence) are often ignored or not considered because of methodological restrictions. With our study, we hope to inspire ecologists to define possible pathways of hierarchical trait influence *a priori* and to adjust their experiments and measurement protocols accordingly. This is not easily done, as resolving the process hierarchy requires the extra effort of quantifying component processes (e.g. species-specific mortality, growth or recruitment) and intermediate ecosystem states (e.g. stand structure or resource limitation). However, failure to do so precludes a mechanistic understanding of biodiversity–ecosystem functioning relationships. Our study also emphasizes the highly dynamic nature of the trait influence on ecosystem functions. This calls for long-term studies. If funding schemes do not allow this, at least the successional stage of the community should be precisely characterized and great caution should be exercised when generalizing the results.

## Supplementary Material

Appendix A. Ecosystem Model. (PDF)

## Supplementary Material

Appendix B. Species Traits and References. (XLSX)

## Supplementary Material

Appendix B. Species Traits and References. (PDF)

## Supplementary Material

Appendix C. Path Model Equations. (PDF)

## Supplementary Material

Appendix D. Figures and Tables. (PDF)

## Supplementary Material

Appendix E. Analysis Code and data for path analysis. (PDF)

## Supplementary Material

Appendix E. Analysis Code and data for path analysis. (CSV)

## Data accessibility

The datasets and analysis code supporting this article have been uploaded as part of the electronic supplementary material.
