# Supplementary material for: Taking a closer look: disentangling effects of functional diversity on ecosystem functions with a trait-based model across hierarchy and time
Source: R Soc Open Sci. 2015 Mar 25;2(3):140541. doi: 10.1098/rsos.140541 (PMC4448832; doi:10.1098/rsos.140541)
Supplement: Appendix A. Ecosystem Model. (PDF) [file rsos140541supp1.pdf]

Appendix A – Ecosystem Model

Changes to the core version of LPJ–GUESS 2.1 (cf. Ahlström et al. 2012)

Disturbance & Mortality

The module of wind-throw is adapted from Lagergren et al. (2012) with adaption of the intensity to fit observations from Holzwarth et al. (2013). Additionally we introduced crushing mortality as a result from wind-throw and other mortality events. This process was fitted to data from Holzwarth et al. (2013). On the other hand, generic random disturbances were dropped from the model. The general concept of patch disturbance – which led to obligatory running of the establishment routine (which would otherwise be called only every 5 years to reduce number of cohorts and thus computational time) and a resetting of the calculation of PAR (photosynthetically active radiation) that reaches the forest floor, such that establishment would not be delayed in that patch – was maintained in that it was evoked when at least 50% of the crown cover in a patch was lost due to mortality (major mortality event).

Wind-throw equations:

$$\text{Windload} \sim \textit{Lognormal}(\mu = -0.14, \text{sd} = 0.54)$$

Drawn annually for each patch with a mean ≈ 1.0 and an imposed interval [0.33, 3.0].

$$\text{Height-Index} = (\text{Height} - \text{Height}_{\text{patch}})/30 + 1$$

$$\text{Allometry-Index} = (\text{Height} - 5) / (\text{Diameter}*100 + 12)$$

Both indices limited to be ≥ 0.

$$\text{Storm-Susceptibility} = \text{Height-Index} * \text{Allometry-Index} * k_{\text{Storm-Susceptibility}}$$

$$MR_{\text{Storm}} = \text{Windload} * \text{Storm-Susceptibility} * m$$

With m = 7.0 (scaling parameter, in Lagergren *et al.* (2012) m = 9.35), $k_{\text{Storm-Susceptibility}}$ the species specific trait characterizing intrinsic susceptibility to wind-throw (between 0 and 1, for most hard-wood species around 0.1) and MR = mortality rate (unitless). Where applicable, units are [m] and metrics refer to the individual if not stated otherwise. The mortality rate was restricted to be ≤ 1.

Crushing equation:

$$MR_{\text{Crushing}} = (0.9*M_{\text{Storm}} + 0.4*(M_{\text{Shade}} + M_{\text{Senescensce}})) / BM$$

With MR = mortality rate (unitless), M = Mortality and BM = biomass, per patch basis, all units [kg C/m^2] and MR_{Crushing} strikes all individuals irrespective of their characteristics.

Crown architecture

In the original model, a tree crown started always from the floor, without a crown-free bole, although the concept was implemented. We set the bole height and thus the height where the crown starts to

53% of the height across all species. This figure was derived from a linear regression on hardwood trees from the Hainich area (Anonymous 2013).

Allometry – maximum height

In the original code the tree height was calculated with:

$$Height = Cmass_{Sapwood} * k_{latosa} / Cmass_{Leaf} / SLA / Wood\ density$$

There was no trait-based upper limit and we deem $Height_{Max}$ to be an ecologically important species specific trait, so that we wanted to include it. We thus modified the formula, such that a maximum height would not be surpassed and this would be attained only asymptotically. We thus changed the formula for height by reducing the species specific constant for leaf area to sapwood cross-sectional area (k_{latosa}) for the individual by a factor *f* depending on the ratio of suggested height (equation above) to $Height_{Max}$:

$$Height\text{-}ratio = Height / Height_{Max}$$

$$f = (1 - \log(\exp((1 - Height\text{-}ratio) * K_{HeightMax}) + 1) / K_{HeightMax}) / Height\text{-}ratio$$

$$k_{latosa}\ (individual) = k_{latosa}\ (species) * f$$

with $K_{HeightMax}$ set to 18, such that there the growth limitation sets on just before attaining maximum height but still being smooth. With this new value of k_{latosa} the new height would be calculated. In this case, e.g., a suggested Height-ratio of 1.0 would lead to a final Height-ratio of 0.98.

Establishment interval

In the original model the establishment routine was called only every 5 years. This was to reduce computational time, such that only every 5 years a new cohort could come into life, whilst maintaining that this interval was short enough to allow for realistic age distributions and forest dynamics. We changed this procedure to get a finer resolution for the initial establishment phase and after disturbances such that we forced the routine to be called every year for the first ten years after patch initialization and after major mortality events and thereafter every 5 years.

Output of LAI and aNPP before Vegetation Dynamics

We let the output of the stand-states of LAI and annual NPP to happen before the vegetation dynamics (establishment, growth and mortality) would be called, because we thought they should reflect what happened with the live trees of the actual period. We think that this reflects the intention of the programmers but it was not correctly implemented.

Grassy understory

We did not simulate shrubs or grass as to just concentrate on the tree species.

Environmental Drivers

The soil-texture was fine-medium, thus with an intermediately high plant available water capacity. Climate data came from the Climatic Research Unit (New et al. 1999), and we used a repeated series from the years 1901 to 1950. Thus environmental drivers were the same for every run.

References

Ahlström A, Schurgers G, Arneth A, Smith B (2012) Robustness and uncertainty in terrestrial ecosystem carbon response to CMIP5 climate change projections. *Environmental Research Letters* 7 (4): 44008.

Anonymous (2013) The Role of Biodiversity for Biogeochemical Cycles and Biotic Interactions in Temperate Deciduous Forests. DFG Graduiertenkolleg 1086. Raw Data. Available: www.uni-goettingen.de/en/82664.html

Holzwarth F, Kahl A, Bauhus J, Wirth C (2013) Many ways to die–partitioning tree mortality dynamics in a near-natural mixed deciduous forest. *Journal of Ecology* 101 (1), 220–230.

Lagergren F, Jönsso AM, Blennow K, Smith B (2012) Implementing storm damage in a dynamic vegetation model for regional applications in Sweden. *Ecological Modelling* 247, 71–82.

New M, Hulme M, Jones P (1999) Representing Twentieth-Century Space--Time Climate Variability. Part I: Development of a 1961--90 Mean Monthly Terrestrial Climatology. *Journal of Climate* 12 (3).
