# Supplementary material for: Taking a closer look: disentangling effects of functional diversity on ecosystem functions with a trait-based model across hierarchy and time
Source: R Soc Open Sci. 2015 Mar 25;2(3):140541. doi: 10.1098/rsos.140541 (PMC4448832; doi:10.1098/rsos.140541)
Supplement: Appendix B. Species Traits and References. (PDF) [file rsos140541supp3.pdf]

Appendix B – References

Allometry

1. Abdul-Hamid H, Mencuccini M (2009) Age- and size-related changes in physiological characteristics and chemical composition of *Acer pseudoplatanus* and *Fraxinus excelsior* trees. Tree Physiology 29 (1): 27–38.
2. Aguinagalde I, Hampe A, Mohanty A, Martín JP, Duminil J et al. (2005) Effects of life-history traits and species distribution on genetic structure at maternally inherited markers in European trees and shrubs. Journal of Biogeography 32 (2): 329–339.
3. Alberti G, Candido P, Peressotti A, Turco S, Piussi P et al. (2005) Aboveground biomass relationships for mixed ash (*Fraxinus excelsior* L. and *Ulmus glabra* Hudson) stands in Eastern Prealps of Friuli Venezia Giulia (Italy). Annals of Forest Science 62 (8): 831–836.
4. Anonymous: Biodiversity Exploratories - Exploratories for large-scale and long-term functional biodiversity research. DFG Infrastruktur-Schwerpunktprogramm 1374. Raw Data. Available: www.biodiversity-exploratories.de. Accessed March 2014.
5. Anonymous: The Role of Biodiversity for Biogeochemical Cycles and Biotic Interactions in Temperate Deciduous Forests. DFG Graduiertenkolleg 1086. Raw Data. Available: www.uni-goettingen.de/en/82664.html. Accessed March 2014.
6. Anonymous: TreeDivNet. A unique global experimental platform for biodiversity and ecosystem research in forests. Site Kreinitz (Germany) - Raw Data. Available: http://www.treedivnet.ugent.be/index.html. Accessed March 2014.
7. Atkinson MD, Atkinson E (2002) Biological Flora of the British Isles: *Sambucus nigra* L. Journal of Ecology 90 (5): 895–923. Available: http://dx.doi.org/10.1046/j.1365-2745.2002.00698.x.
8. Bader MK, Siegwolf R, Körner C (2010) Sustained enhancement of photosynthesis in mature deciduous forest trees after 8 years of free air CO_2 enrichment. Planta 232 (5): 1115–1125.
9. Bartelink HH (1996) Allometric relationships on biomass and needle area of Douglas-fir. Forest Ecology and Management 86 (1): 193–203.
10. Bartelink HH (1997) Allometric relationships for biomass and leaf area of beech (*Fagus sylvatica* L) 1.
11. Brzeziecki B, Kienast F (1994) Classifying the life-history strategies of trees on the basis of the Grimian model. Forest Ecology and Management 69 (1): 167–187.
12. Čermák J (1998) Leaf distribution in large trees and stands of the floodplain forest in southern Moravia. Tree Physiology 18 (11): 727–737.
13. Ďurkovič J, Čaňová I, Priwitzer T, Biroščíková M, Kapraľ P et al. (2010) Field assessment of photosynthetic characteristics in micropropagated and grafted wych elm (*Ulmus glabra* Huds.) trees. Plant Cell, Tissue and Organ Culture (PCTOC) 101 (2): 221–228.
14. Gower ST, Reich PB, Son Y (1993) Canopy dynamics and aboveground production of five tree species with different leaf longevities. Tree Physiology 12 (4): 327–345.
15. Harbinson J, Woodward FI (1984) Field measurements of the gas exchange of woody plant species in simulated sunflecks. Annals of Botany 53 (6): 841–851.

16. Hester AJ, Millard P, Baillie GJ, Wendler R (2004) How does timing of browsing affect above-and below-ground growth of *Betula pendula*, *Pinus sylvestris* and *Sorbus aucuparia*. Oikos 105 (3): 536–550.
17. Hölscher D (2004) Leaf traits and photosynthetic parameters of saplings and adult trees of co-existing species in a temperate broad-leaved forest. Basic and Applied Ecology 5 (2): 163–172.
18. Köcher P, Gebauer T, Horna V, Leuschner C (2009) Leaf water status and stem xylem flux in relation to soil drought in five temperate broad-leaved tree species with contrasting water use strategies. Annals of Forest Science 66 (1): 1.
19. Landsberg J, Mäkelä A, Sievänen R, Kukkola M (2005) Analysis of biomass accumulation and stem size distributions over long periods in managed stands of *Pinus sylvestris* in Finland using the 3-PG model. Tree Physiology 25 (7): 781–792.
20. Le Goff N, Granier A, Ottorini J, Peiffer M (2004) Biomass increment and carbon balance of ash (*Fraxinus excelsior*) trees in an experimental stand in northeastern France. Annals of Forest Science 61 (6): 577–588.
21. Lichtenthaler HK, Ač A, Marek MV, Kalina J, Urban O (2007) Differences in pigment composition, photosynthetic rates and chlorophyll fluorescence images of sun and shade leaves of four tree species. Plant Physiology and Biochemistry 45 (8): 577–588. Available: http://www.sciencedirect.com/science/article/pii/S0981942807000939.
22. Martínez-Vilalta J, Vanderklein D, Mencuccini M (2007) Tree height and age-related decline in growth in Scots pine (*Pinus sylvestris* L.). Oecologia 150 (4): 529–544.
23. Nagel R, Spellmann H (2008) Growth, treatment and yield of pure beech (*Fagus sylvatica* L.) stands in northwest Germany. In: Nordwestdeutsche Forstliche Versuchsanstalt (NW-FVA), editor. Ergebnisse angewandter Forschung zur Buche. Göttingen. pp. 221–265.
24. Nikolić NP, Krstić BĐ, Pajević SP, Orlović SS (2006) Variability of leaf characteristics in different pedunculate oak genotypes (*Ouercus robur* L.). Zbornik Matice srpske za prirodne nauke (111): 95–105.
25. Pigott CD (1991) Biological Flora of the British Isles: *Tilia cordata* Miller. The Journal of Ecology: 1147–1207.
26. Raspé O, Findlay C, Jacquemart A (2000) Biological Flora of the British Isles: *Sorbus aucuparia* L. Journal of Ecology 88 (5): 910–930.
27. Reich PB, Ellsworth DS, Walters MB, Vose JM, Gresham C et al. (1999) Generality of leaf trait relationships: a test across six biomes. Ecology 80 (6): 1955–1969.
28. Roloff A (2008) Tree of the year 2007: Scots Pine (*Pinus sylvestris* L.). Biology, Ecology, Uses, Diseases. In: Nordwestdeutsche Forstliche Versuchsanstalt (NW-FVA), editor. Die Waldkiefer. Göttingen. pp. 15–31.
29. Roloff A, Weisgerber H, Lang U, Stimm B, Schütt P (2008) Enzyklopädie der Holzgewächse: Wiley-VCH.
30. Rouifed S, Handa IT, David J, Hättenschwiler S (2010) The importance of biotic factors in predicting global change effects on decomposition of temperate forest leaf litter. Oecologia 163 (1): 247–256.
31. Silfver T, Mikola J, Rousi M, Roininen H, Oksanen E (2007) Leaf litter decomposition differs among genotypes in a local *Betula pendula* population. Oecologia 152 (4): 707–714.
32. Thomas PA, Polwart A (2003) Biological Flora of the British Isles: *Taxus baccata* L. Journal of Ecology 91 (3): 489–524.

33. Uri V, Lõhmus K, Ostonen I, Tullus H, Vildo M (2007) Biomass production, foliar and root characteristics and nutrient accumulation in young silver birch (*Betula pendula* Roth.) stand growing on abandoned agricultural land. European journal of forest research 126 (4): 495–506.
34. van Hees AF, Bartelink HH (1993) Needle area relationships of Scots pine in the Netherlands. Forest Ecology and Management 58 (1): 19–31.
35. Vieilledent G, Courbaud B, Kunstler G, Dhôte J, Clark JS (2010) Individual variability in tree allometry determines light resource allocation in forest ecosystems: a hierarchical Bayesian approach. Oecologia 163 (3): 759–773.
36. Vincke C, Breda N, Granier A, Devillez F (2005) Evapotranspiration of a declining *Quercus robur* (L.) stand from 1999 to 2001. I. Trees and forest floor daily transpiration. Annals of Forest Science 62 (6): 503–512.
37. Withington JM, Reich PB, Oleksyn J, Eissenstat DM (2006) Comparisons of structure and life span in roots and leaves among temperate trees. Ecological Monographs 76 (3): 381–397.
38. Wright IJ, Reich PB, Westoby M, Ackerly DD, Baruch Z et al. (2004) The worldwide leaf economics spectrum. Nature 428 (6985): 821–827.
39. Zotz G, Pepin S, Körner C (2005) No Down-Regulation of Leaf Photosynthesis in Mature Forest Trees after Three Years of Exposure to Elevated CO_2. Plant Biology 7 (4): 369–374.

Climate

1. Bittner S, Talkner U, Krämer I, Beese F, Hölscher D et al. (2010) Modeling stand water budgets of mixed temperate broad-leaved forest stands by considering variations in species specific drought response. Agricultural and forest meteorology 150 (10): 1347–1357.
2. Linder M (2000) Developing adaptive forest management strategies to cope with climate change. Tree Physiology 20 (5-6): 299–307.
3. Murray MB, Cannell MG, Smith RI (1989) Date of budburst of fifteen tree species in Britain following climatic warming. Journal of Applied Ecology: 693–700.
4. Pigott CD (1991) Biological Flora of the British Isles: *Tilia cordata* Miller. The Journal of Ecology: 1147–1207.
5. Roloff A, Weisgerber H, Lang U, Stimm B, Schütt P (2008) Enzyklopädie der Holzgewächse: Wiley-VCH.
6. Sykes MT, Prentice IC, Cramer W (1996) A bioclimatic model for the potential distributions of north European tree species under present and future climates. Journal of Biogeography: 203–233.
7. USDA N (2014) The PLANTS Database. Available: http://plants.usda.gov, 7 March 2014.
8. Walentowski H, Ewald J (2003) Die Rolle der Schwarzerle in den Pflanzengesellschaften Mitteleuropas. In: Bayerische Landesanstalt für Wald und Forstwirtschaft (LWF), editor. Beiträge zur Schwarzerle. Freising. pp. 11–19.

CN-leaf

1. Aas G (2003) Die Schwarzerle, *Alnus glutinosa.* Dendrologische Anmerkungen. In: Bayerische Landesanstalt für Wald und Forstwirtschaft (LWF), editor. Beiträge zur Schwarzerle. Freising. pp. 7–10.

2. Anonymous: The Role of Biodiversity for Biogeochemical Cycles and Biotic Interactions in Temperate Deciduous Forests. DFG Graduiertenkolleg 1086. Raw Data. Available: www.uni-goettingen.de/en/82664.html. Accessed March 2014.
3. Gower ST, Reich PB, Son Y (1993) Canopy dynamics and aboveground production of five tree species with different leaf longevities. Tree Physiology 12 (4): 327–345.
4. Kattge J, Diaz S, Lavorel S, Prentice IC, Leadley P et al. (2011) TRY–a global database of plant traits. Global Change Biology 17 (9): 2905–2935.
5. Kazda M, Salzer J, Reiter I (2000) Photosynthetic capacity in relation to nitrogen in the canopy of a *Quercus robur*, *Fraxinus angustifolia* and *Tilia cordata* flood plain forest. Tree Physiology 20 (15): 1029–1037.
6. Nikolić NP, Krstić BĐ, Pajević SP, Orlović SS (2006) Variability of leaf characteristics in different pedunculate oak genotypes (*Ouercus robur* L.). Zbornik Matice srpske za prirodne nauke (111): 95–105.
7. Nordén U (1994) Leaf litterfall concentrations and fluxes of elements in deciduous tree species. Scandinavian Journal of Forest Research 9 (1-4): 9–16.
8. Pigott CD (1991) Biological Flora of the British Isles: *Tilia cordata* Miller. The Journal of Ecology: 1147–1207.
9. Reich PB, Ellsworth DS, Walters MB, Vose JM, Gresham C et al. (1999) Generality of leaf trait relationships: a test across six biomes. Ecology 80 (6): 1955–1969.
10. Roloff A, Weisgerber H, Lang U, Stimm B, Schütt P (2008) Enzyklopädie der Holzgewächse: Wiley-VCH.
11. Rouifed S, Handa IT, David J, Hättenschwiler S (2010) The importance of biotic factors in predicting global change effects on decomposition of temperate forest leaf litter. Oecologia 163 (1): 247–256.
12. Silfver T, Mikola J, Rousi M, Roininen H, Oksanen E (2007) Leaf litter decomposition differs among genotypes in a local *Betula pendula* population. Oecologia 152 (4): 707–714.
13. Uri V, Lõhmus K, Ostonen I, Tullus H, Vildo M (2007) Biomass production, foliar and root characteristics and nutrient accumulation in young silver birch (*Betula pendula* Roth.) stand growing on abandoned agricultural land. European journal of forest research 126 (4): 495–506.
14. Withington JM, Reich PB, Oleksyn J, Eissenstat DM (2006) Comparisons of structure and life span in roots and leaves among temperate trees. Ecological Monographs 76 (3): 381–397.
15. Zotz G, Pepin S, Körner C (2005) No Down-Regulation of Leaf Photosynthesis in Mature Forest Trees after Three Years of Exposure to Elevated CO_2. Plant Biology 7 (4): 369–374.

CN-root

1. Burton A, Pregitzer KS, Ruess R, Hendrick R, Allen M (2002) Root respiration in North American forests: effects of nitrogen concentration and temperature across biomes. Oecologia 131 (4): 559–568.
2. Pregitzer KS, Kubiske ME, Yu CK, Hendrick RL (1997) Relationships among root branch order, carbon, and nitrogen in four temperate species. Oecologia 111 (3): 302–308.

CN-wood

1. Kattge J, Diaz S, Lavorel S, Prentice IC, Leadley P et al. (2011) TRY–a global database of plant traits. Global Change Biology 17 (9): 2905–2935.
2. Keller R, Nussbaumer T (1993) Bestimmung des Stickstoffgehalts von Holz und Holzwerkstoffen mittels Oxidation und Chemilumineszenz-Detektion von Stickstoffmonoxid. Holz als Roh-und Werkstoff 51 (1): 21–26.

Crownarea max

1. Anonymous: The Role of Biodiversity for Biogeochemical Cycles and Biotic Interactions in Temperate Deciduous Forests. DFG Graduiertenkolleg 1086. Raw Data. Available: www.uni-goettingen.de/en/82664.html. Accessed March 2014.
2. Pigott CD (1991) Biological Flora of the British Isles: *Tilia cordata* Miller. The Journal of Ecology: 1147–1207.
3. Roloff A, Weisgerber H, Lang U, Stimm B, Schütt P (2008) Enzyklopädie der Holzgewächse: Wiley-VCH.

Drought Tolerance

1. Niinemets Ü, Valladares F (2006) Tolerance to shade, drought, and waterlogging of temperate Northern Hemisphere trees and shrubs. Ecological Monographs 76 (4): 521–547.

Establishment

1. Kleyer M, Bekker RM, Knevel IC, Bakker JP, Thompson K et al. (2008) The LEDA Traitbase: a database of life-history traits of the Northwest European flora. Journal of Ecology 96 (6): 1266–1274.

Fire Resistance

1. USDA N (2014) The PLANTS Database. Available: http://plants.usda.gov, 7 March 2014.

k_latosa (Leaf Area to Sapwood cross sectional area relationship)

1. Abdul-Hamid H, Mencuccini M (2009) Age- and size-related changes in physiological characteristics and chemical composition of *Acer pseudoplatanus* and *Fraxinus excelsior* trees. Tree Physiology 29 (1): 27–38.
2. Alberti G, Candido P, Peressotti A, Turco S, Piussi P et al. (2005) Aboveground biomass relationships for mixed ash (*Fraxinus excelsior* L. and *Ulmus glabra* Hudson) stands in Eastern Prealps of Friuli Venezia Giulia (Italy). Annals of Forest Science 62 (8): 831–836.
3. Aranda I, Gil L, Pardos JA (2005) Seasonal changes in apparent hydraulic conductance and their implications for water use of European beech (*Fagus sylvatica* L.) and sessile oak [*Quercus petraea* (Matt.) Liebl] in South Europe. Plant Ecology 179 (2): 155–167.
4. Arneth A, Kelliher FM, Bauer G, Hollinger DY, Byers JN et al. (1996) Environmental regulation of xylem sap flow and total conductance of *Larix gmelinii* trees in eastern Siberia. Tree Physiology 16 (1-2): 247–255.
5. Atkinson MD, Atkinson E (2002) Biological Flora of the British Isles: *Sambucus nigra* L. Journal of Ecology 90 (5): 895–923. Available: http://dx.doi.org/10.1046/j.1365-2745.2002.00698.x.

6. Barbaroux C, Bréda N (2002) Contrasting distribution and seasonal dynamics of carbohydrate reserves in stem wood of adult ring-porous sessile oak and diffuse-porous beech trees. Tree Physiology 22 (17): 1201–1210.
7. Barnard DM, Meinzer FC, Lachenbruch B, McCulloh KA, Johnson DM et al. (2011) Climate-related trends in sapwood biophysical properties in two conifers: avoidance of hydraulic dysfunction through coordinated adjustments in xylem efficiency, safety and capacitance. Plant, Cell & Environment 34 (4): 643–654.
8. Bartelink HH (1996) Allometric relationships on biomass and needle area of Douglas-fir. Forest Ecology and Management 86 (1): 193–203.
9. Bartelink HH (1997) Allometric relationships for biomass and leaf area of beech (*Fagus sylvatica* L) 1.
10. Bartelink HH (2000) Effects of stand composition and thinning in mixed-species forests: a modeling approach applied to Douglas-fir and beech. Tree Physiology 20 (5-6): 399–406.
11. Bovard BD, Curtis PS, Vogel CS, Su H, Schmid HP (2005) Environmental controls on sap flow in a northern hardwood forest. Tree Physiology 25 (1): 31–38.
12. Bréda N, Granier A, Aussenac G (1995) Effects of thinning on soil and tree water relations, transpiration and growth in an oak forest (*Quercus petraea* (Matt.) Liebl.). Tree Physiology 15 (5): 295–306.
13. Büntgen U, Esper J, Schmidhalter M, Frank DC, Treydte K et al. (2004) Using recent and historical larch wood to build a 1300-year Valais-chronology. Trace 2: 85–92.
14. Burger H (1949) Holz, Blattmenge und Zuwachs: Die Buche. Mitt Schweiz Anst Forstl Versuchsw. 26: 419–468.
15. Čermák J (1998) Leaf distribution in large trees and stands of the floodplain forest in southern Moravia. Tree Physiology 18 (11): 727–737.
16. Davi H, Barbaroux C, Dufrêne E, Francois C, Montpied P et al. (2008) Modelling leaf mass per area in forest canopy as affected by prevailing radiation conditions. ecological modelling 211 (3): 339–349.
17. David TS, Ferreira MI, Cohen S, Pereira JS, David JS (2004) Constraints on transpiration from an evergreen oak tree in southern Portugal. Agricultural and forest meteorology 122 (3): 193–205.
18. Duursma RA, Kolari P, Perämäki M, Pulkkinen M, Mäkelä A et al. (2009) Contributions of climate, leaf area index and leaf physiology to variation in gross primary production of six coniferous forests across Europe: a model-based analysis. Tree Physiology 29 (5): 621–639.
19. Edwards WR (1986) Precision weighing lysimetry for trees, using a simplified tared-balance design. Tree Physiology 1 (2): 127–144.
20. Gebauer T, Horna V, Leuschner C (2008) Variability in radial sap flux density patterns and sapwood area among seven co-occurring temperate broad-leaved tree species. Tree Physiology 28 (12): 1821–1830.
21. Genet H, Bréda N, Dufrêne E (2010) Age-related variation in carbon allocation at tree and stand scales in beech (*Fagus sylvatica* L.) and sessile oak (*Quercus petraea* (Matt.) Liebl.) using a chronosequence approach. Tree Physiology 30 (2): 177–192.
22. Haneca K, Boeren I, van Acker J, Beeckman H (2006) Dendrochronology in suboptimal conditions: tree rings from medieval oak from Flanders (Belgium) as dating tools and archives of past forest management. Vegetation history and archaeobotany 15 (2): 137–144.

23. Herbst M, Roberts JM, Rosier PTW, Taylor ME, Gowing DJ (2007) Edge effects and forest water use: A field study in a mixed deciduous woodland. Forest Ecology and Management 250 (3): 176–186.
24. Hölscher D (2004) Leaf traits and photosynthetic parameters of saplings and adult trees of co-existing species in a temperate broad-leaved forest. Basic and Applied Ecology 5 (2): 163–172.
25. Hölscher D, Koch O, Korn S, Leuschner C (2005) Sap flux of five co-occurring tree species in a temperate broad-leaved forest during seasonal soil drought. Trees 19 (6): 628–637.
26. Johansson T (2007) Biomass production and allometric above-and below-ground relations for young birch stands planted at four spacings on abandoned farmland. Forestry 80 (1): 41–52.
27. Kaufmann MR, Troendle CA (1981) The relationship of leaf area and foliage biomass to sapwood conducting area in four subalpine forest tree species. Forest Science 27 (3): 477–482.
28. Köcher P, Gebauer T, Horna V, Leuschner C (2009) Leaf water status and stem xylem flux in relation to soil drought in five temperate broad-leaved tree species with contrasting water use strategies. Annals of Forest Science 66 (1): 1.
29. Kodama N, Barnard RL, Salmon Y, Weston C, Ferrio JP et al. (2008) Temporal dynamics of the carbon isotope composition in a *Pinus sylvestris* stand: from newly assimilated organic carbon to respired carbon dioxide. Oecologia 156 (4): 737–750.
30. Kort I de (1986) Wood structure and growth ring width of vital and non-vital Douglas fir (*Pseudotsuga menziesii*) from a single stand in the Netherlands. IAWA Bull 7: 309–318.
31. Köstner B, Falge E, Tenhunen JD (2002) Age-related effects on leaf area/sapwood area relationships, canopy transpiration and carbon gain of Norway spruce stands (*Picea abies*) in the Fichtelgebirge, Germany. Tree Physiology 22 (8): 567–574.
32. Kuusk A, Kuusk J, Lang M (2009) A dataset for the validation of reflectance models. Remote Sensing of Environment 113 (5): 889–892.
33. Lambs L, Loubiat M, Girel J, Tissier J, Peltier J et al. (2006) Survival and acclimatation of *Populus nigra* to drier conditions after damming of an alpine river, southeast France. Annals of Forest Science 63 (4): 377–385.
34. Magel EA, Drouet A, Claudot AC, Ziegler H (1991) Formation of heartwood substances in the stem of *Robinia pseudoacacia* L. Trees 5 (4): 203–207.
35. Mäkelä A, Virtanen K, Nikinmaa E (1995) The effects of ring width, stem position, and stand density on the relationship between foliage biomass and sapwood area in Scots pine (*Pinus sylvestris*). Canadian journal of forest research 25 (6): 970–977.
36. Martínez-Vilalta J, Sala A, Piñol J (2004) The hydraulic architecture of Pinaceae–a review. Plant Ecology 171 (1-2): 3–13.
37. Martínez-Vilalta J, Vanderklein D, Mencuccini M (2007) Tree height and age-related decline in growth in Scots pine (*Pinus sylvestris* L.). Oecologia 150 (4): 529–544.
38. McDowell N, Barnard H, Bond B, Hinckley T, Hubbard R et al. (2002) The relationship between tree height and leaf area: sapwood area ratio. Oecologia 132 (1): 12–20.

39. Mencuccini M, Grace J (1996) Hydraulic conductance, light interception and needle nutrient concentration in Scots pine stands and their relations with net primary productivity. Tree Physiology 16 (5): 459–468.
40. Miller RF, Eddleman LE, Angell RF (1987) Relationship of western juniper stem conducting tissue and basal circumference to leaf area and biomass. Western North American Naturalist 47 (3): 349–354.
41. Neuenschwander T (2008) Climate sensitivity of a millennial-long *Larix decidua* tree-ring chronology in the French Maritime Alps.
42. Niklas KJ (1997) Mechanical properties of black locust (*Robinia pseudoacacia* L.) wood. Size-and age-dependent variations in sap-and heartwood. Annals of Botany 79 (3): 265–272.
43. Nocetti M, Brunetti M, Ducci F, Romagnoli M, Santi F (2010) Variability of wood properties in two wild cherry clonal trials. Wood science and technology 44 (4): 621–637.
44. O'Hara KL, Lähde E, Laiho O, Norokorpi Y, Saksa T (1999) Leaf area and tree increment dynamics on a fertile mixed-conifer site in southern Finland. Annals of Forest Science 56 (3): 237–247.
45. Oren R, Werk KS, Schulze E (1986) Relationships between foliage and conducting xylem in *Picea abies* (L.) Karst. Trees 1 (1): 61–69.
46. Pausch RC, Grote EE, Dawson TE (2000) Estimating water use by sugar maple trees: considerations when using heat-pulse methods in trees with deep functional sapwood. Tree Physiology 20 (4): 217–227.
47. Poyatos R, Čermák J, Llorens P (2007) Variation in the radial patterns of sap flux density in pubescent oak (*Quercus pubescens*) and its implications for tree and stand transpiration measurements. Tree Physiology 27 (4): 537–548.
48. Roloff A, Weisgerber H, Lang U, Stimm B, Schütt P (2008) Enzyklopädie der Holzgewächse: Wiley-VCH.
49. Ruehr NK, Knohl A, Buchmann N (2010) Environmental variables controlling soil respiration on diurnal, seasonal and annual time-scales in a mixed mountain forest in Switzerland. Biogeochemistry 98 (1-3): 153–170.
50. Sala A, Carey EV, Callaway RM (2001) Dwarf mistletoe affects whole-tree water relations of Douglas fir and western larch primarily through changes in leaf to sapwood ratios. Oecologia 126 (1): 42–52.
51. Sampson DA, Janssens IA, Curiel Yuste J, Ceulemans R (2007) Basal rates of soil respiration are correlated with photosynthesis in a mixed temperate forest. Global Change Biology 13 (9): 2008–2017.
52. Savill PS, Kanowski PJ, Gourlay ID, Jarvis AR (1993) Short note: genetic and intra-tree variation in the number of sapwood rings in *Quercus robur* and *Q. petraea.* Silvae genetica 42 (1993): 371.
53. Schäfer KV, Oren R, Tenhunen JD (2000) The effect of tree height on crown level stomatal conductance. Plant, Cell & Environment 23 (4): 365–375.
54. Sellin A (1996) Sapwood amount in *Picea abies* (L.) Karst. determined by tree age and radial growth rate. Holzforschung-International Journal of the Biology, Chemistry, Physics and Technology of Wood 50 (4): 291–296.
55. Sellin A, Kupper P (2006) Spatial variation in sapwood area to leaf area ratio and specific leaf area within a crown of silver birch. Trees 20 (3): 311–319.

56. Shelburne VB, Hedden RL, Allen RM (1993) The effect of site, stand density, and sapwood permeability on the relationship between leaf area and sapwood area in loblolly pine (*Pinus taeda* L.). Forest Ecology and Management 58 (3): 193–209.
57. Sohar K, Vitas A, Läänelaid A (2012) Sapwood estimates of pedunculate oak (*Quercus robur* L.) in eastern Baltic. Dendrochronologia 30 (1): 49–56.
58. Soudani K, François C, Le Maire G, Le Dantec V, Dufrêne E (2006) Comparative analysis of IKONOS, SPOT, and ETM+ data for leaf area index estimation in temperate coniferous and deciduous forest stands. Remote Sensing of Environment 102 (1): 161–175.
59. Sterck FJ, Zweifel R, Sass-Klaassen U, Chowdhury Q (2008) Persisting soil drought reduces leaf specific conductivity in Scots pine (*Pinus sylvestris*) and pubescent oak (*Quercus pubescens*). Tree Physiology 28 (4): 529–536.
60. Uri V, Lõhmus K, Ostonen I, Tullus H, Vildo M (2007) Biomass production, foliar and root characteristics and nutrient accumulation in young silver birch (*Betula pendula* Roth.) stand growing on abandoned agricultural land. European journal of forest research 126 (4): 495–506.
61. van Hees AF, Bartelink HH (1993) Needle area relationships of Scots pine in the Netherlands. Forest Ecology and Management 58 (1): 19–31.
62. Vanninen P, Ylitalo H, Sievänen R, Mäkelä A (1996) Effects of age and site quality on the distribution of biomass in Scots pine (*Pinus sylvestris* L.). Trees 10 (4): 231–238.
63. Vincke C, Breda N, Granier A, Devillez F (2005) Evapotranspiration of a declining *Quercus robur* (L.) stand from 1999 to 2001. I. Trees and forest floor daily transpiration. Annals of Forest Science 62 (6): 503–512.
64. Waring RH, Schroeder PE, Oren R (1982) Application of the pipe model theory to predict canopy leaf area. Canadian journal of forest research 12 (3): 556–560.
65. Whitehead D (1978) The estimation of foliage area from sapwood basal area in Scots pine. Forestry 51 (2): 137–149.
66. Widlowski JL, Verstraete MM, Pinty B, Gobron N (2003) Allometric relationships of selected European tree species. Rep. EUR 20855 EN.
67. Zianis D, Seura SM (2005) Biomass and stem volume equations for tree species in Europe: Finnish Society of Forest Science, Finnish Forest Research Institute.

Leaf longevity

1. Defila C (2003) Phytophenological observations in the Grisons: Trends of phytophenological time series. Schweizerische Zeitschrift fur Forstwesen 154 (8): 333–339.
2. Gower ST, Reich PB, Son Y (1993) Canopy dynamics and aboveground production of five tree species with different leaf longevities. Tree Physiology 12 (4): 327–345.
3. Harbinson J, Woodward FI (1984) Field measurements of the gas exchange of woody plant species in simulated sunflecks. Annals of Botany 53 (6): 841–851.
4. Kikuzawa K (1982) Leaf survival and evolution in Betulaceae. Annals of Botany 50 (3): 345–353.
5. Lauschee F (1987) Analysen des Jahresverlaufs der Belaubung von Bäumen und Sträuchern.

6. Mediavilla S, Escudero A (2003) Photosynthetic capacity, integrated over the lifetime of a leaf, is predicted to be independent of leaf longevity in some tree species. New phytologist 159 (1): 203–211.
7. Reich PB, Ellsworth DS, Walters MB, Vose JM, Gresham C et al. (1999) Generality of leaf trait relationships: a test across six biomes. Ecology 80 (6): 1955–1969.
8. Rogers RW, Clifford HT (1993) The taxonomic and evolutionary significance of leaf longevity. New phytologist 123 (4): 811–821.
9. Roloff A, Weisgerber H, Lang U, Stimm B, Schütt P (2008) Enzyklopädie der Holzgewächse: Wiley-VCH.
10. Schütt P, Schuck HJ, Stimm B (1992) Lexikon der Forstbotanik: Morphologie, Pathologie, Ökologie und Systematik wichtiger Baum-und Straucharten: ecomed.
11. Thomas PA, El-Barghati M, Polwart A (2007) Biological Flora of the British Isles: *Juniperus communis* L. Journal of Ecology 95 (6): 1404–1440. Available: http://dx.doi.org/10.1111/j.1365-2745.2007.01308.x.
12. Thomas PA, Polwart A (2003) Biological Flora of the British Isles: *Taxus baccata* L. Journal of Ecology 91 (3): 489–524.
13. Withington JM, Reich PB, Oleksyn J, Eissenstat DM (2006) Comparisons of structure and life span in roots and leaves among temperate trees. Ecological Monographs 76 (3): 381–397.
14. Wright IJ, Reich PB, Westoby M, Ackerly DD, Baruch Z et al. (2004) The worldwide leaf economics spectrum. Nature 428 (6985): 821–827.

Longevity

1. Atkinson MD, Atkinson E (2002) Biological Flora of the British Isles: *Sambucus nigra* L. Journal of Ecology 90 (5): 895–923. Available: http://dx.doi.org/10.1046/j.1365-2745.2002.00698.x.
2. Brzeziecki B, Kienast F (1994) Classifying the life-history strategies of trees on the basis of the Grimian model. Forest Ecology and Management 69 (1): 167–187.
3. Heinz Utschig (2003) Waldwachstumskundliche Charakterisierung der Schwarzerle (*Alnus glutinosa* (L.) GAERTNER) am Beispiel der Wuchsreihe Wasserburg 642. In: Bayerische Landesanstalt für Wald und Forstwirtschaft (LWF), editor. Beiträge zur Schwarzerle. Freising. pp. 23–26.
4. Holzwarth F, Kahl A, Bauhus J, Wirth C (2013) Many ways to die–partitioning tree mortality dynamics in a near-natural mixed deciduous forest. Journal of Ecology 101 (1): 220–230.
5. Morin X, Fahse L, Scherer-Lorenzen M, Bugmann H (2011) Tree species richness promotes productivity in temperate forests through strong complementarity between species. Ecology letters 14 (12): 1211–1219.
6. Roloff A (2008) Tree of the year 2007: Scots Pine (*Pinus sylvestris* L.). Biology, Ecology, Uses, Diseases. In: Nordwestdeutsche Forstliche Versuchsanstalt (NW-FVA), editor. Die Waldkiefer. Göttingen. pp. 15–31.
7. Roloff A, Weisgerber H, Lang U, Stimm B, Schütt P (2008) Enzyklopädie der Holzgewächse: Wiley-VCH.
8. Thomas PA, El-Barghati M, Polwart A (2007) Biological Flora of the British Isles: *Juniperus communis* L. Journal of Ecology 95 (6): 1404–1440. Available: http://dx.doi.org/10.1111/j.1365-2745.2007.01308.x.

Root Distribution

1. Aas G (2002) Die Gewöhnliche Esche (*Fraxinus excelsior*). Dendrologische Anmerkungen. In: Bayerische Landesanstalt für Wald und Forstwirtschaft (LWF), editor. Beiträge zur Esche. Freising. pp. 1–5.

2. Aas G (2003) Die Schwarzerle, *Alnus glutinosa.* Dendrologische Anmerkungen. In: Bayerische Landesanstalt für Wald und Forstwirtschaft (LWF), editor. Beiträge zur Schwarzerle. Freising. pp. 7–10.
3. Albrecht L (2010) Waldbauliche Erfahrungen mit der Vogelkirsche. In: Bayerische Landesanstalt für Wald und Forstwirtschaft (LWF), editor. Beiträge zur Vogelkirsche. Freising. pp. 24–33.
4. Asche N (1999) Bodenschutzkalkung und das Wurzelsystem der Rotbuche (*Fagus Sylvatica*) Eine Fallstudie im Sauerland. Forstwissenschaftliches Centralblatt vereinigt mit Tharandter forstliches Jahrbuch 118 (1-6): 294–301.
5. Bréda N, Granier A, Barataud F, Moyne C (1995) Soil water dynamics in an oak stand. Plant and Soil 172 (1): 17–27.
6. Dobrowolska D, Hein S, Oosterbaan A, Wagner S, Clark J et al. (2011) A review of European ash (*Fraxinus excelsior* L.): implications for silviculture. Forestry 84 (2): 133–148.
7. Gulder H (1996) Das Wurzelwerk der Hainbuche. In: Bayerische Landesanstalt für Wald und Forstwirtschaft (LWF), editor. Beiträge zur Hainbuche. Freising. pp. 30–37.
8. Hinckley TM, Duhme F, Hinckley AR, Richter H (1983) Drought relations of shrub species: assessment of the mechanisms of drought resistance. Oecologia 59 (2-3): 344–350.
9. Hruska J, Čermák J, Šustek S (1999) Mapping tree root systems with ground-penetrating radar. Tree Physiology 19 (2): 125–130.
10. Kreutzer K (1961) Wurzelbildung junger Waldbäume auf Pseudogleyböden. Forstwissenschaftliches Centralblatt 80 (11): 356–392.
11. Kutschera L, Lichtenegger E (1997) Wurzeln. Bewurzelung von Pflanzen in Verschiedenen Lebensräumen. Stapfia 49.
12. Nordmann B (2009) Wurzelwachstum des Bergahorns. In: Bayerische Landesanstalt für Wald und Forstwirtschaft (LWF), editor. Beiträge zum Bergahorn. Freising. pp. 30–32.
13. Pigott CD (1991) Biological Flora of the British Isles: *Tilia cordata* Miller. The Journal of Ecology: 1147–1207.
14. Roloff A (2008) Tree of the year 2007: Scots Pine (*Pinus sylvestris* L.). Biology, Ecology, Uses, Diseases. In: Nordwestdeutsche Forstliche Versuchsanstalt (NW-FVA), editor. Die Waldkiefer. Göttingen. pp. 15–31.
15. Schütt P, Schuck HJ, Stimm B (1992) Lexikon der Forstbotanik: Morphologie, Pathologie, Ökologie und Systematik wichtiger Baum-und Straucharten: ecomed.
16. Thomas PA, Polwart A (2003) Biological Flora of the British Isles: *Taxus baccata* L. Journal of Ecology 91 (3): 489–524.
17. Walentowski H, Kölling C, Ewald J (2007) Die Waldkiefer – bereit für den Klimawandel. In: Bayerische Landesanstalt für Wald und Forstwirtschaft (LWF), editor. Beiträge zur Waldkiefer. Freising. pp. 37–43.

Shade Tolerance

1. Niinemets Ü, Valladares F (2006) Tolerance to shade, drought, and waterlogging of temperate Northern Hemisphere trees and shrubs. Ecological Monographs 76 (4): 521–547.

SLA (specific leaf area)

1. Abdul-Hamid H, Mencuccini M (2009) Age- and size-related changes in physiological characteristics and chemical composition of *Acer pseudoplatanus* and *Fraxinus excelsior* trees. Tree Physiology 29 (1): 27–38.
2. Aguinagalde I, Hampe A, Mohanty A, Martín JP, Duminil J et al. (2005) Effects of life-history traits and species distribution on genetic structure at maternally inherited markers in European trees and shrubs. Journal of Biogeography 32 (2): 329–339.
3. Alberti G, Candido P, Peressotti A, Turco S, Piussi P et al. (2005) Aboveground biomass relationships for mixed ash (*Fraxinus excelsior* L. and *Ulmus glabra* Hudson) stands in Eastern Prealps of Friuli Venezia Giulia (Italy). Annals of Forest Science 62 (8): 831–836.
4. Anonymous: TreeDivNet. A unique global experimental platform for biodiversity and ecosystem research in forests. Site Kreinitz (Germany) - Raw Data. Available: http://www.treedivnet.ugent.be/index.html. Accessed March 2014.
5. Atkinson MD, Atkinson E (2002) Biological Flora of the British Isles: *Sambucus nigra* L. Journal of Ecology 90 (5): 895–923. Available: http://dx.doi.org/10.1046/j.1365-2745.2002.00698.x.
6. Bader MK, Siegwolf R, Körner C (2010) Sustained enhancement of photosynthesis in mature deciduous forest trees after 8 years of free air CO_2 enrichment. Planta 232 (5): 1115–1125.
7. Bartelink HH (1996) Allometric relationships on biomass and needle area of Douglas-fir. Forest Ecology and Management 86 (1): 193–203.
8. Bartelink HH (1997) Allometric relationships for biomass and leaf area of beech (*Fagus sylvatica* L) 1.
9. Čermák J (1998) Leaf distribution in large trees and stands of the floodplain forest in southern Moravia. Tree Physiology 18 (11): 727–737.
10. Ďurkovič J, Čaňová I, Priwitzer T, Biroščíková M, Kapraľ P et al. (2010) Field assessment of photosynthetic characteristics in micropropagated and grafted wych elm (*Ulmus glabra* Huds.) trees. Plant Cell, Tissue and Organ Culture (PCTOC) 101 (2): 221–228.
11. Gower ST, Reich PB, Son Y (1993) Canopy dynamics and aboveground production of five tree species with different leaf longevities. Tree Physiology 12 (4): 327–345.
12. Harbinson J, Woodward FI (1984) Field measurements of the gas exchange of woody plant species in simulated sunflecks. Annals of Botany 53 (6): 841–851.
13. Hester AJ, Millard P, Baillie GJ, Wendler R (2004) How does timing of browsing affect above-and below-ground growth of *Betula pendula*, *Pinus sylvestris* and *Sorbus aucuparia*. Oikos 105 (3): 536–550.
14. Hölscher D (2004) Leaf traits and photosynthetic parameters of saplings and adult trees of co-existing species in a temperate broad-leaved forest. Basic and Applied Ecology 5 (2): 163–172.
15. Kazda M, Salzer J, Reiter I (2000) Photosynthetic capacity in relation to nitrogen in the canopy of a *Quercus robur*, *Fraxinus angustifolia* and *Tilia cordata* flood plain forest. Tree Physiology 20 (15): 1029–1037.
16. Köcher P, Gebauer T, Horna V, Leuschner C (2009) Leaf water status and stem xylem flux in relation to soil drought in five temperate broad-leaved tree species with contrasting water use strategies. Annals of Forest Science 66 (1): 1.

17. Landsberg J, Mäkelä A, Sievänen R, Kukkola M (2005) Analysis of biomass accumulation and stem size distributions over long periods in managed stands of *Pinus sylvestris* in Finland using the 3-PG model. Tree Physiology 25 (7): 781–792.
18. Le Goff N, Granier A, Ottorini J, Peiffer M (2004) Biomass increment and carbon balance of ash (*Fraxinus excelsior*) trees in an experimental stand in northeastern France. Annals of Forest Science 61 (6): 577–588.
19. Lichtenthaler HK, Ač A, Marek MV, Kalina J, Urban O (2007) Differences in pigment composition, photosynthetic rates and chlorophyll fluorescence images of sun and shade leaves of four tree species. Plant Physiology and Biochemistry 45 (8): 577–588. Available: http://www.sciencedirect.com/science/article/pii/S0981942807000939.
20. Martínez-Vilalta J, Vanderklein D, Mencuccini M (2007) Tree height and age-related decline in growth in Scots pine (*Pinus sylvestris* L.). Oecologia 150 (4): 529–544.
21. Nikolić NP, Krstić BĐ, Pajević SP, Orlović SS (2006) Variability of leaf characteristics in different pedunculate oak genotypes (*Ouercus robur* L.). Zbornik Matice srpske za prirodne nauke (111): 95–105.
22. Reich PB, Ellsworth DS, Walters MB, Vose JM, Gresham C et al. (1999) Generality of leaf trait relationships: a test across six biomes. Ecology 80 (6): 1955–1969.
23. Rouifed S, Handa IT, David J, Hättenschwiler S (2010) The importance of biotic factors in predicting global change effects on decomposition of temperate forest leaf litter. Oecologia 163 (1): 247–256.
24. Silfver T, Mikola J, Rousi M, Roininen H, Oksanen E (2007) Leaf litter decomposition differs among genotypes in a local *Betula pendula* population. Oecologia 152 (4): 707–714.
25. Uri V, Lõhmus K, Ostonen I, Tullus H, Vildo M (2007) Biomass production, foliar and root characteristics and nutrient accumulation in young silver birch (*Betula pendula* Roth.) stand growing on abandoned agricultural land. European journal of forest research 126 (4): 495–506.
26. van Hees AF, Bartelink HH (1993) Needle area relationships of Scots pine in the Netherlands. Forest Ecology and Management 58 (1): 19–31.
27. Withington JM, Reich PB, Oleksyn J, Eissenstat DM (2006) Comparisons of structure and life span in roots and leaves among temperate trees. Ecological Monographs 76 (3): 381–397.
28. Wright IJ, Reich PB, Westoby M, Ackerly DD, Baruch Z et al. (2004) The worldwide leaf economics spectrum. Nature 428 (6985): 821–827.
29. Zotz G, Pepin S, Körner C (2005) No Down-Regulation of Leaf Photosynthesis in Mature Forest Trees after Three Years of Exposure to Elevated CO_2. Plant Biology 7 (4): 369–374.

Wood density

1. Kattge J, Diaz S, Lavorel S, Prentice IC, Leadley P et al. (2011) TRY–a global database of plant traits. Global Change Biology 17 (9): 2905–2935.
2. Martin Čulík (2009) Black elder wood for Slovak folk wind musical instruments making. Proceedings of the ACOUSTICS High Tatras 2009 “34th International Acoustical Conference - EAA Symposium”.
3. Wagenführ R (2000) Holzatlas. Leipzig: Carl Hanser Verlag.

4. Wirth C, Ogle K, Kattge J, Nöllert S, Boenisch G, Nüske A, Zhang R, Jian N, Frank D, Knauer M, Sartor K, Garvie K, Kahl A FET - Functional Ecology of Trees Database Project. Available: bgc-jena.mpg.de/bgc-organisms/pmwiki.php/Research/FET.
5. Zanne AE, Lopez-Gonzalez G, Coomes DA, Ilic J, Jansen S et al. (2009) Global wood density database. Dryad. Identifier: http://hdl. handle. net/10255/dryad 235.
