# Supplementary material for: Taking a closer look: disentangling effects of functional diversity on ecosystem functions with a trait-based model across hierarchy and time
Source: R Soc Open Sci. 2015 Mar 25;2(3):140541. doi: 10.1098/rsos.140541 (PMC4448832; doi:10.1098/rsos.140541)
Supplement: Appendix C. Path Model Equations. (PDF) [file rsos140541supp4.pdf]

Appendix C – Path Model Equations

The path model consists of a number of mathematical and regression equations. In the terminology of path analysis variables that have an arrow pointing to them are endogenous variables, they are to be calculated or to be estimated. Endogenous variables may also point to other variables, when they are used to calculate or estimate them. Exogenous variables only point to other variables but have no arrows pointing on them. Exogenous variables are the metrics of functional composition (FC), all other are endogenous. Additionally, all endogenous variables denote only the biodiversity effect in htem, indicated by a preceding delta sign (Δx). Cf. Methods in the main document and Figure C1 in this document.

Some relationships are mathematical equations, they are derived directly from the code of the vegetation model.

ΔABC (biodiversity effect in annual biomass change) is the sum of four subordinate rates:

$$\Delta ABC = \Delta Growth + \Delta Recruitment - \Delta Turnover - \Delta Mortality$$

With: Growth = increment in BM, Recruitment= growth of new regeneration, Turnover = turnover of roots and leaves, Mortality = mortality of live trees, each [kg $C/m^2/a$].

Growth is net primary production (NPP) reduced by what is set off for reproduction (each [kg $C/m^2/a$]):

$$\Delta Growth = \Delta NPP - \Delta Reproduction$$

Reproduction is set as a fixed fraction of NPP:

$$\Delta Reproduction = 0.1 * \Delta NPP$$

And GPP is gross primary production reduced by autotrophic respiration ($Resp_A$), each [kg $C/m^2/a$]:

$$\Delta NPP = \Delta GPP - \Delta Resp_A$$

Mortality is the sum of five distinct mortality processes (Shade, Senescence, Storm, Fire and Crushing, each [kg $C/m^2/a$]):

$$\Delta Mortality = \Delta M_{Shade} + \Delta M_{Senescence} + \Delta M_{Storm} + \Delta M_{Fire} + \Delta M_{Crushing}$$

Some relationships are rather tight but not exact relationships in terms of equations or units, they are also derived from the code of the vegetation model:

$$\Delta GPP \sim \Delta LAI, \Delta LAI_{SD}\ \Delta WS$$

$$\Delta M_{Crushing} \sim \Delta M_{Shade}, \Delta M_{Senescence}, \Delta M_{Storm}$$

With: LAI the leaf area index [m^2/m^2], mean weighted by patch BM, LAI_{SD} the patch-BM weighted temporal and spatial population standard deviation of LAI across patches and years (of the respective time periods), and WS = Water stress, a metric between 0 and 1 indicating water stress in a patch.

The path model is further made up by a series of statistical models that relate the above input variables directly and indirectly via intermediate steps to the measures of functional composition (FC). Here FC stands for measures for three facets of functional composition: functional richness, functional dissimilarity and functional identity.

$\Delta BM \sim FC$

$\Delta Height \sim FC$

$\Delta Height_{SD} \sim FC$

$\Delta LAI_{SD} \sim FC$

$\Delta LAI \sim FC, \Delta BM, \Delta Height, \Delta Height_{SD}$

$\Delta WS \sim FC, \Delta LAI$

$\Delta Resp_{A} \sim FC, \Delta GPP, \Delta BM$

$\Delta M_{Shade} \sim FC, \Delta LAI, \Delta BM$

$\Delta M_{Senescence} \sim FC, \Delta BM$

$\Delta M_{Storm} \sim FC, \Delta BM, \Delta Height$

$\Delta M_{Fire} \sim FC, \Delta BM, \Delta WS$

$\Delta Recruitment \sim FC, \Delta LAI, \Delta Reproduction$

$\Delta Turnover \sim FC, \Delta LAI, \Delta LAI_{SD}$

With: BM = vegetation Biomass [kg C/m^2], Height = tree height [m], $Height_{SD}$ = population standard deviation of height [m], LAI = leaf area index [m^2/m^2, LAI_{SD} the patch-BM weighted temporal and spatial population standard deviation of LAI across patches and years (of the respective time periods), WS = Water stress, a metric between 0 and 1 indicating water stress in a patch; GPP = gross primary production, $Resp_A$ = autotrophic respiration, each [kg C/m^2/a]. All indices were, where applicable, averages weighted by tree BM or patch BM.

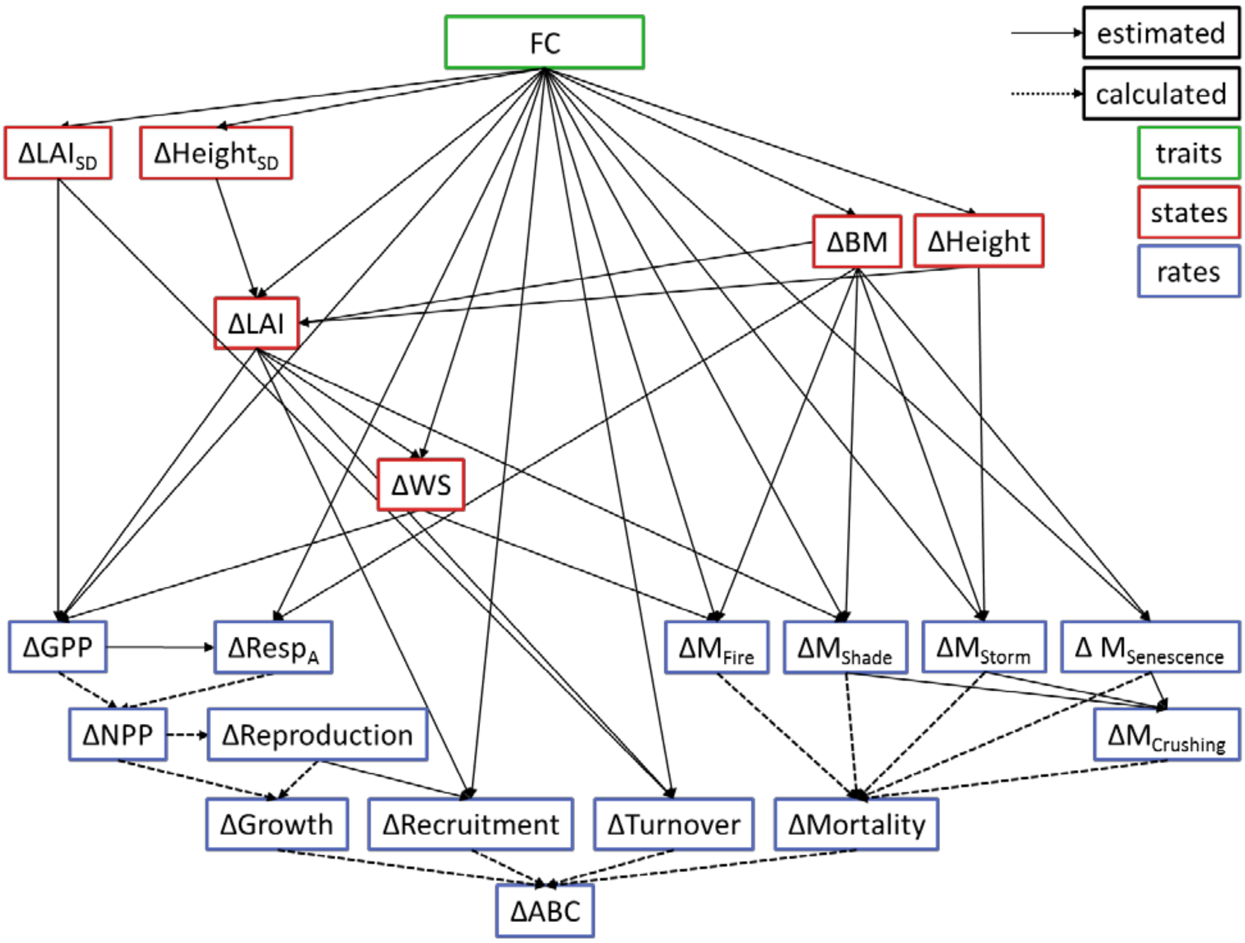

Figure C1. Path model used to analyse the effect paths of functional composition (FC) on the target variable ΔABC (biodiversity effect in annual biomass change) via intermediate states and rates. All states and rates represent the biodiversity effect in them (difference between observation and null-model expectation), denoted by a leading Δ. Solid lines were estimated with linear regressions as part of the path modelling. Dotted lines were calculated, such that ΔABC was the sum of ΔGrowth, ΔRecruitment, ΔTurnover (leaf and roots) and ΔMortality and ΔMortality was the sum of five mortality processes. FC comprises functional richness, functional dissimilarity and functional identity.

States are: BM = biomass [kg C/m^2], Height = tree height [m], LAI = leaf area index [m^2/m^2], LAI$_{SD}$ the standard deviation of LAI across patches and years in a time period, Height$_{SD}$ the standard deviation of Height in a patch, and WS = a metric between 0 and 1 indicating water stress.

Rates [kg C/m^2/a]: ABC = annual biomass change, Growth= growth of existing trees, Recruitment = growth of new regeneration, Turnover = turnover of roots and leaves, Mortality =mortality of living trees, M = mortality due to five processes: Shade, Senescence, Storm, Fire and Crushing, GPP = gross primary productivity, Resp$_A$ = autotrophic respiration, NPP = net primary productivity, Reproduction = allocation of NPP for Recruitment.

We analysed the biodiversity effect on states and rates, indicated by a leading Δ. Where applicable, we used averages weighted by tree and / or patch biomass.
