# Supplementary material for: Taking a closer look: disentangling effects of functional diversity on ecosystem functions with a trait-based model across hierarchy and time
Source: R Soc Open Sci. 2015 Mar 25;2(3):140541. doi: 10.1098/rsos.140541 (PMC4448832; doi:10.1098/rsos.140541)
Supplement: Appendix D. Figures and Tables. (PDF) [file rsos140541supp5.pdf]

Appendix D – Figures and Tables

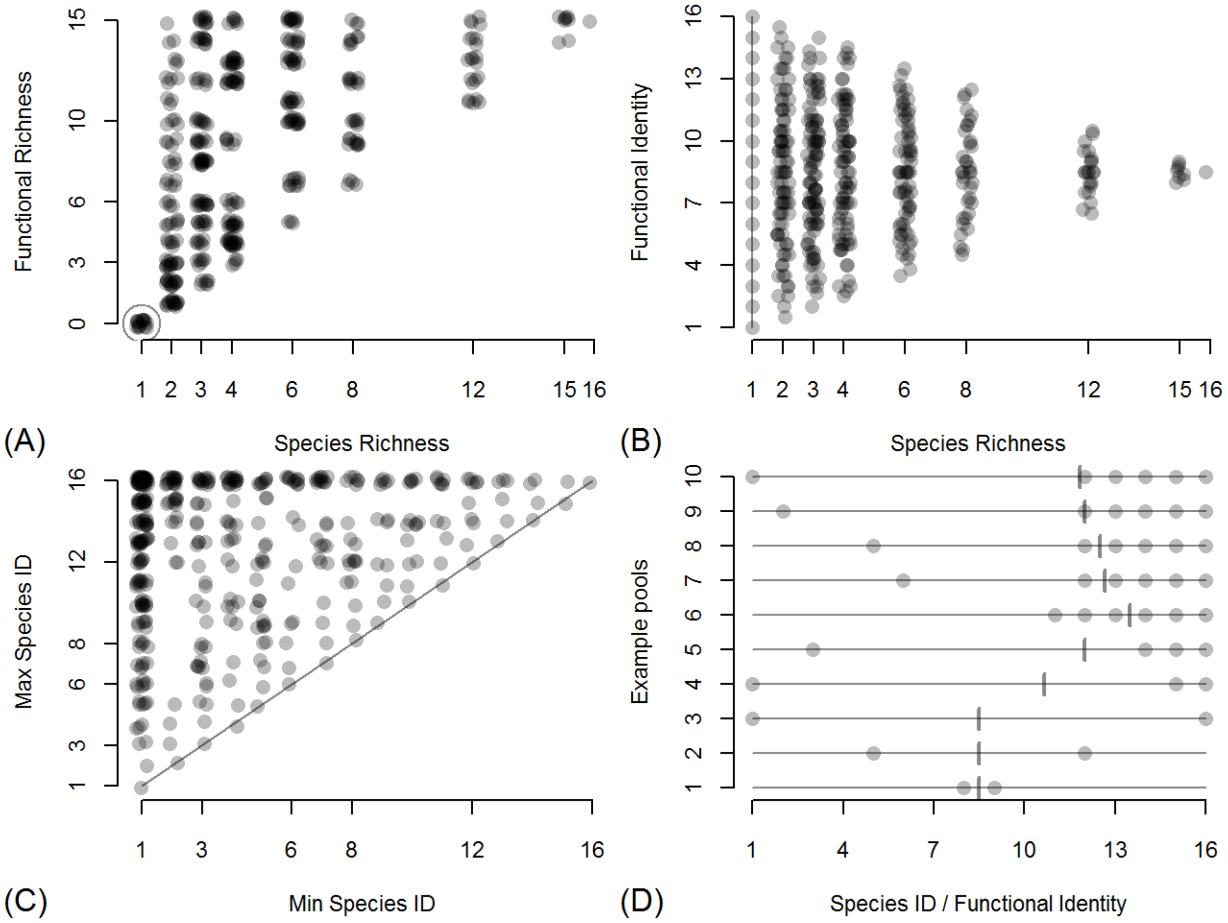

Figure S1. Species pools in the scenarios. Each dot represents one out of 416 scenarios. Functional identity was calculated with equal abundance for each species.

(A) Species richness and functional richness

(B) Species richness and functional identity (unweighted mean of hyper-trait)

(C) Identities of the species with the lowest and highest position on the hyper-trait axis.

(D) Example species pools on horizontal lines, vertical ticks mark the corresponding functional identity (unweighted mean of hyper trait).

In (A) to (C) the 16 single species runs are marked by a surrounding circle or a continuous line and a jitter was added to make overlaying scenarios visible.

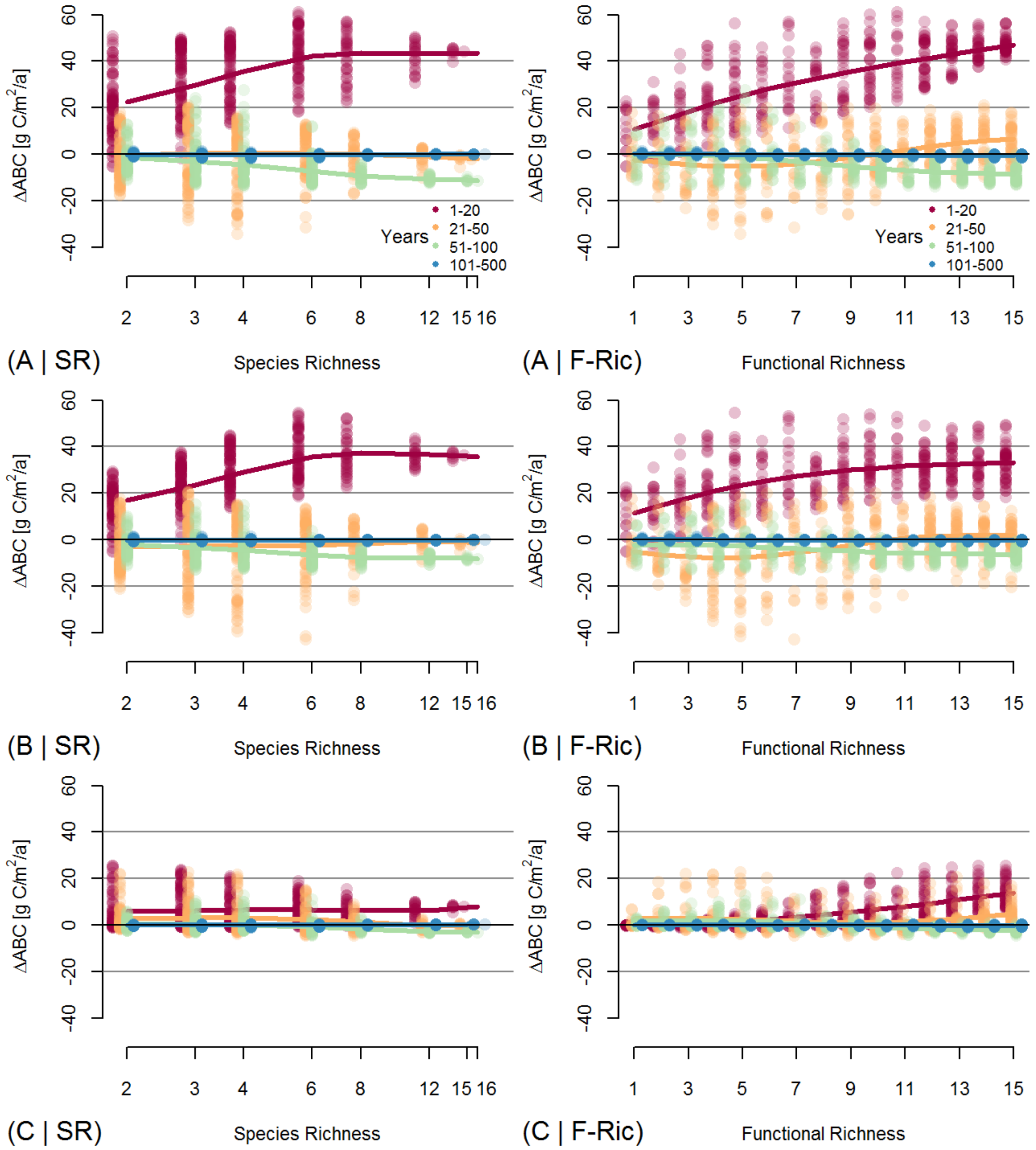

Figure S2. Additive partitioning of biodiversity effect in ABC (ΔABC) over species richness (SR, left) and over functional richness (F-Ric, range of the hyper-trait, right). Following Loreau & Hector (2001), separated for time periods. Each dot represents the average from a scenario run over the time interval of the period with a small shift to enhance visibility. Time periods in different colours, and overlaid by smoothed splines to guide the eye. (A) total, (B) complementarity, (C) selection effects.

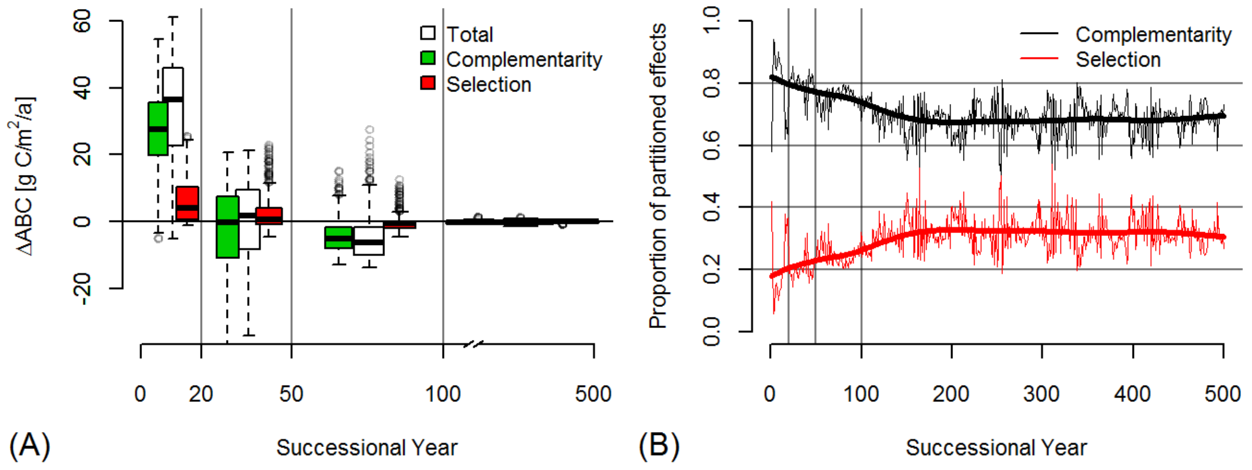

Figure S3. Additive partitioning of biodiversity effect in ABC (ΔABC) over time. Following Loreau & Hector (2001).

(A) The distribution of the total and partitioned biodiversity effects of all 400 scenarios over time periods are displayed in boxplots (median as bold line, hinges as interquartile ranges (IQR) and whiskers extend from there to the extremes or 1.5 times the IQR, whichever is shorter, beyond that single runs as points). White = total, green = complementary, red = selection effects. Vertical lines separate the four different time periods.

(B) Proportion of partitioned effects ($Proportion_{Effect}$) over successional time: $Proportion_{Effect} = |\Delta ABC_{Effect}| / (|\Delta ABC_{Selection}| + |\Delta ABC_{Complementarity}|)$. Smoothed splines as thick lines to guide the eye. Vertical lines separate the four different time periods.

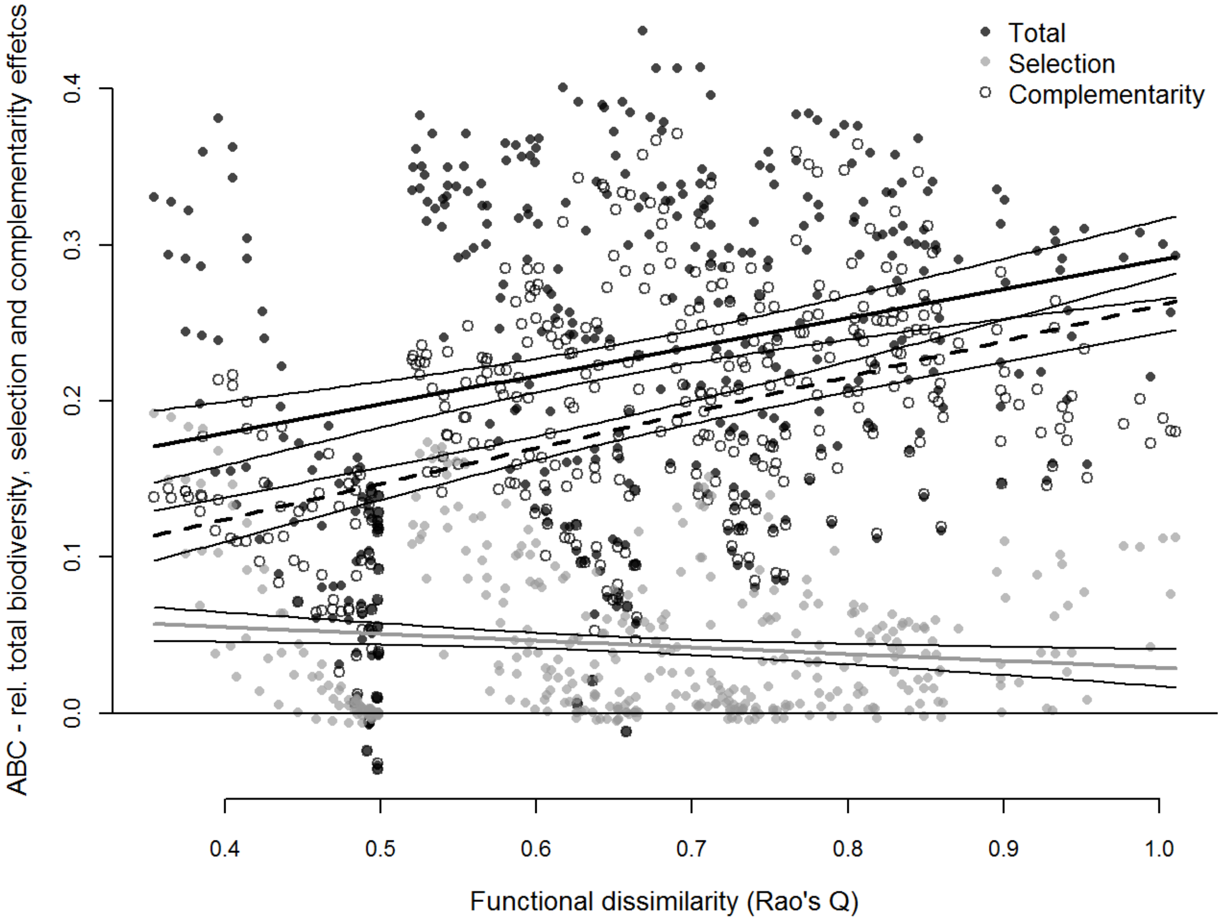

Figure S4-I. Additive partitioning of relative biodiversity effect of ABC (ΔABC) over functional dissimilarity (F-Diss). Following Loreau & Hector (2001). Total relative biodiversity effect in ABC ($\Delta ABC/ABC_{Null}$) (black dots), complementarity effects (open dots) and selection (grey dots) effects over F-Diss (Rao's Q) of all 400 mixture runs from period 1. Lines: linear regression models for effects against F-Diss; relative total biodiversity effect (black, slope=0.19), complementarity (dashed, slope=0.23) and selection (grey, slope=-0.04) with respective 95% confidence intervals. Below also the original figure referred to. Note that functional dispersion (FDis) and F-Diss tend to be very tightly correlated, cf. Laliberté & Legendre (2010). This figure is used to compare our results with results from Morin *et al.* (2011), see the following figure.

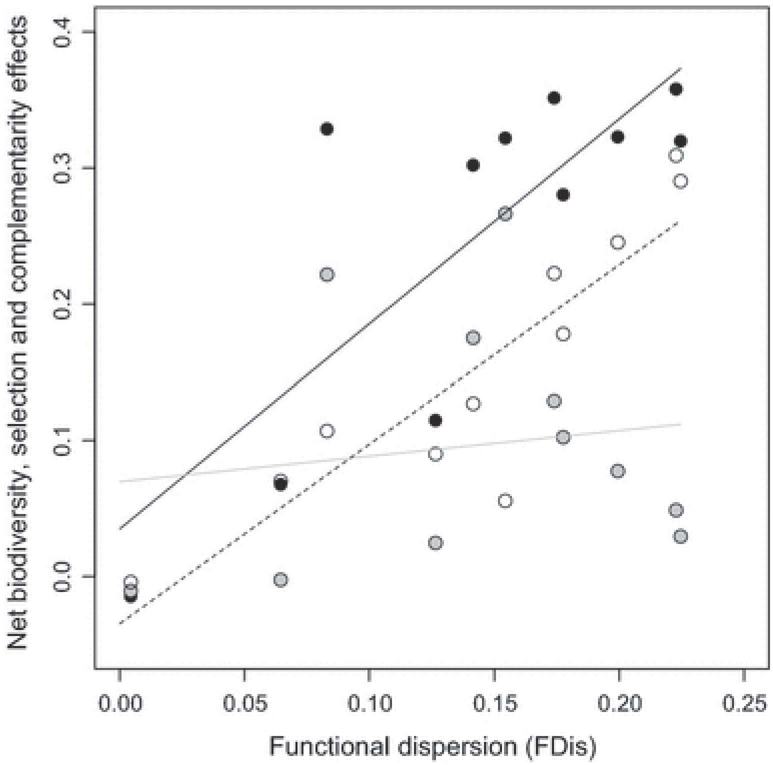

Figure S4-II. From Morin et al. (2011). © 2011 Blackwell Publishing Ltd/CNRS. With the kind permission by the authors and the publishers John Wiley and Sons, License Number: 3464140192156. Original figure legend: "Net biodiversity (black dots), selection (open dots) and complementarity (grey dots) effects as a function of functional dispersion (FDis) index for the simulation with an original richness of 30 species considering all sites together ($n = 11$). The effects were calculated following the original method but divided by the expected forest productivity based on monocultures, and values are square-root transformed to meet the assumptions of the analysis while preserving positive and negative signs. Black plain line: linear regression model for net biodiversity effect against FDis (slope = 2.62, $P < 0.005$); grey plain line: linear regression model for selection effect against FDis (slope = 1.30, $P = 0.162$); dashed line: linear regression model for complementarity effect against FDis (slope = 2.33, $P < 0.001$)." Contrary to the original caption, *selection* effects are not open but *grey dots* and *complementarity* effects are not grey but *open dots* and the both were confused in the original paper (Morin, pers. communication 2014). Unlike in this figure, we referred to the "net" effects as "total" effects.

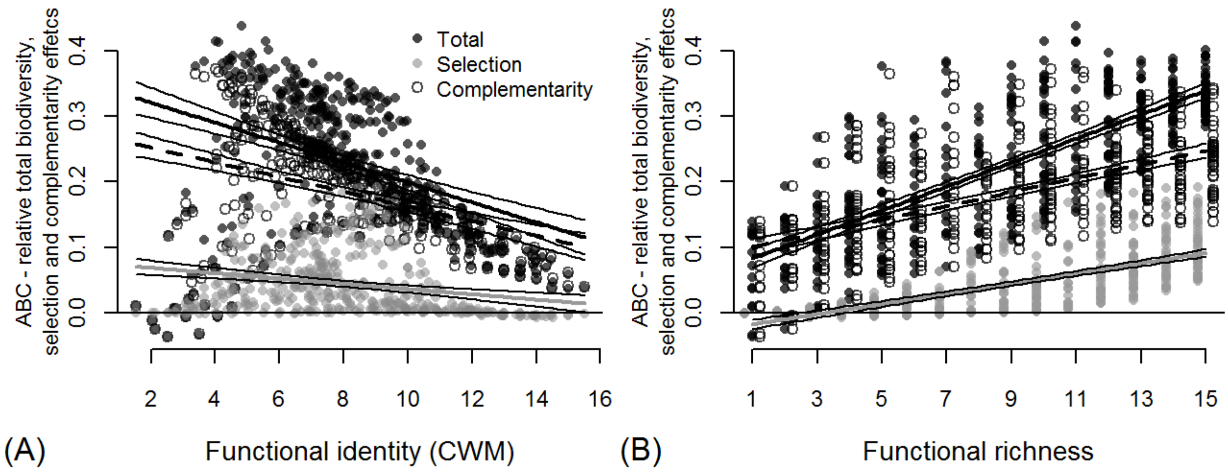

Figure S5. Additive partitioning of relative biodiversity effect in ABC (ΔABC) over functional identity and richness. Following Loreau & Hector (2001). Relative biodiversity effect in ABC ($\Delta ABC/ABC_{Null}$) (black dots), complementarity effects (open dots) and selection (grey dots) effects over functional identity (F-ID, CWM) and functional richness (F-Ric) of all 400 mixture runs from period 1. Lines: linear regression models for effects against F-ID or F-Ric with respective 95% confidence intervals.

(A) F-ID, relative total biodiversity effect (black, slope=-0.015), complementarity (dashed, slope=-0.011) and selection (grey, slope=-0.004).

(B) F-Ric, relative total biodiversity effect (black, slope=0.018), complementarity (dashed, slope=0.011) and selection (grey, slope=0.0077). Dots are slightly shifted to enhance visibility.

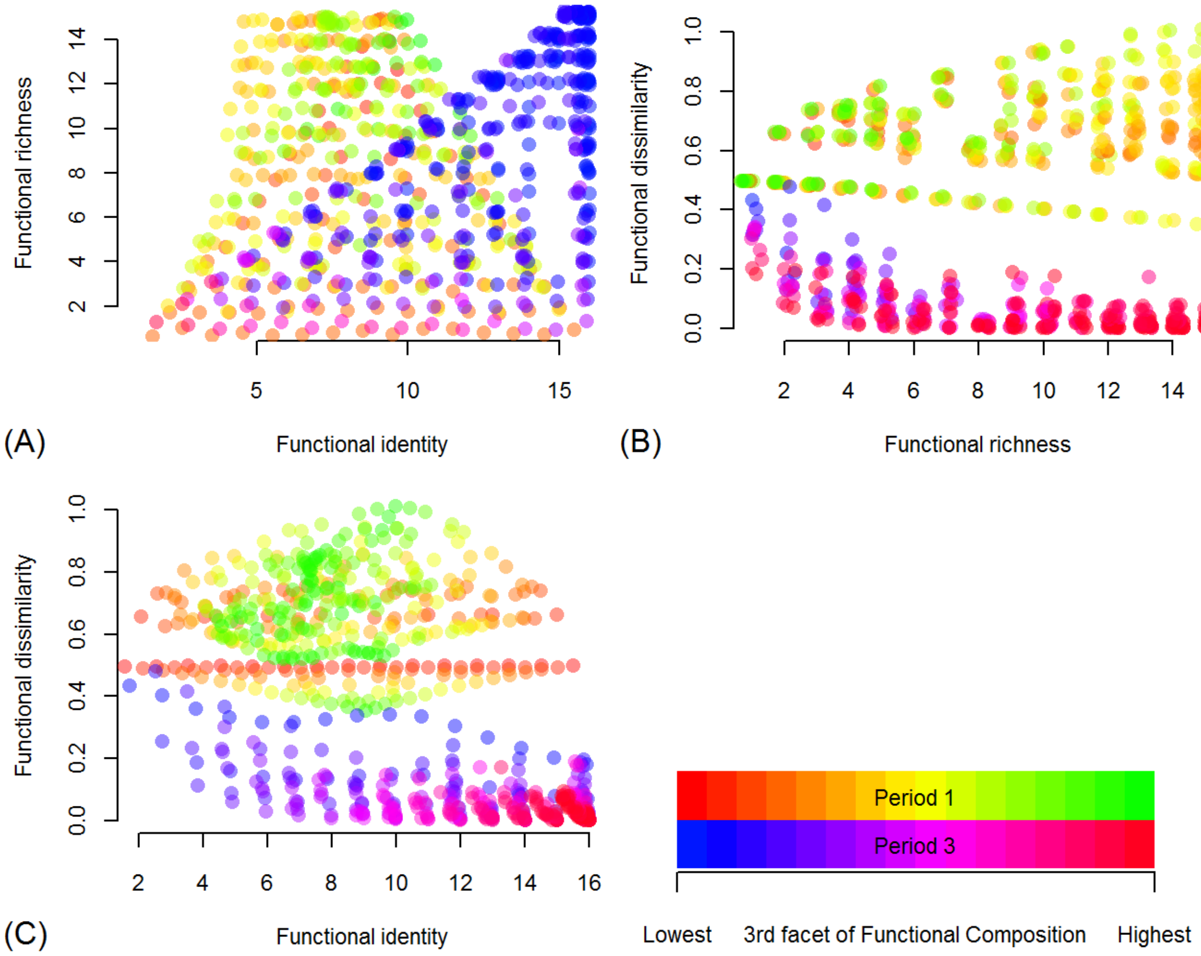

Figure S6. Scatters of facets of realised functional composition (FC) for the time periods 1 and 3. This figure shall illustrate the magnitude of independence between the three facets of FC that were used as predictors. Whilst Fig. 2 and Fig. S1 show the relationship of the three FC-metrics as the scenarios are designed, we see here their realised values during the simulations in two time periods – as such they were used as predictors in the path model. The figures are organised as tri-variate plots with the third facet that is not on an axis represented in a colour gradient. The third facets are: (A) functional dissimilarity (F-Diss), (B) functional identity (F-ID) and (C) functional richness (F-Ric). The colour scale is different for the two periods: period 1 increasing from red to green, period 3 increasing from blue to magenta. In (A) and (B) F-Ric was jittered to enhance visibility. Additionally we report here the Pearson's bi-variate correlation coefficients (Period 1/3) were: F-ID~F-Ric (-0.12/0.62), F-Ric~F-Diss (0.3/-0.62), F-ID~F-Diss (0.04/-0.58).

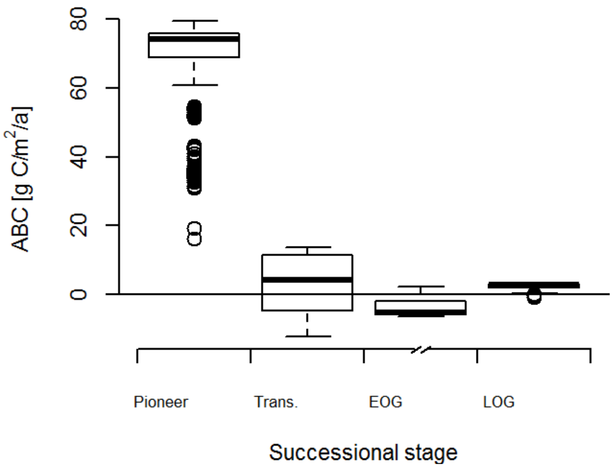

Figure S7-I. Modelled annual biomass change (ABC) of all 400 mixture scenario runs over successional stages. The four successional stages are: ‘*pioneer*’1-100 years, *Trans.* ‘transition’ 101-200 years, *EOG* ‘early old-growth’ 201-400 years and *LOG* ‘late old-growth’ 401-500 years as in Wirth and Lichstein 2009 (LOG is here only 401 to 500 years rather than 401 to 600 years). This figure is used to compare our results with results from Wirth and Lichstein 2009, see the following figure.

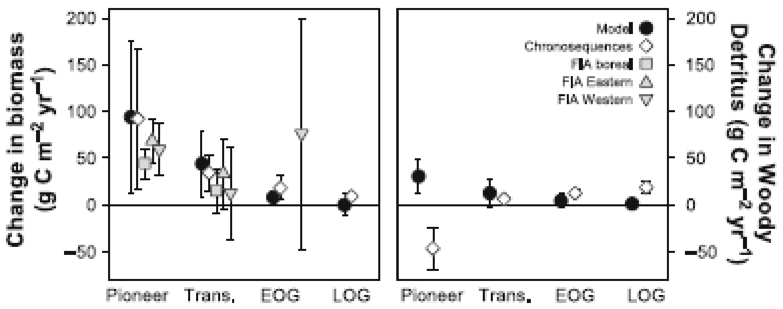

Fig. 5.9 Comparison of modelled and measured changes of aboveground biomass (*left panel*) and coarse woody detritus (*right panel*) in g C m^{-2} $year^{-1}$ within the four successional stages ‘*pioneer*,’ *Trans.* ‘transition’, *EOG* ‘early old-growth’, and *LOG* ‘late old-growth’. *Error bars* standard deviation. The sample unit is a forest sequence. FIA Unites States Forest Inventory and Analysis database (see Chap. 14 by Lichstein et al., this volume)

Figure S7-II. From Wirth and Lichstein (2009). © 2009 Springer Verlag Berlin Heidelberg. With the kind permission by the authors and the publisher Springer Verlag Berlin Heidelberg, License Number: 3464150357296.

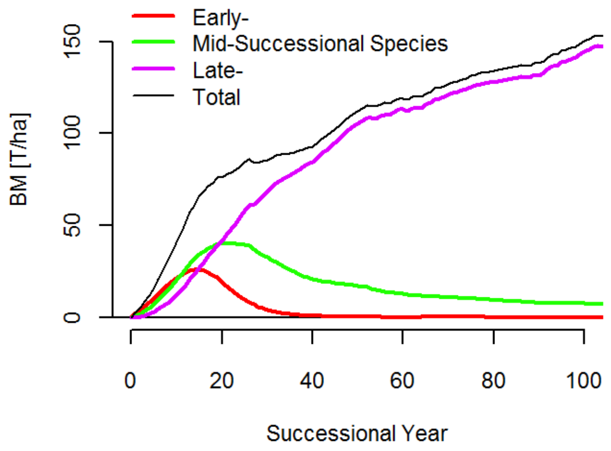

Figure S8-I. Development of biomass split over species successional categories. Modelled biomass (BM) [T/ha] over successional time averaged over 125 mixture scenario runs that included at least one species in each successional category. The categories contained the following species: Early: Species 1-5, Mid: Species 6-12, Late: Species 13-16. Biomass was assumed to have a mass ratio of 50% carbon to make this figure comparable. This figure is used to compare our results with results from Kinzig and Pacala (2001), see the following figure.

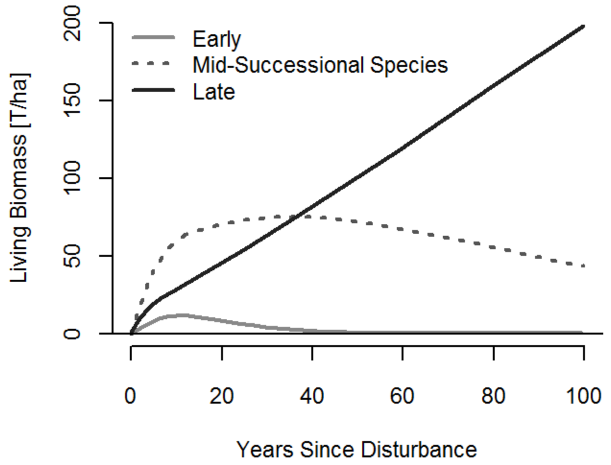

Figure S8-II. Adaption of Figure 9.1 from Kinzig and Pacala (2001). Original figure legend: "Steady-state relationship between living biomass and years since disturbance for a landscape containing all three species in Case II."

Table S1. Number of mixture scenario runs per combination of species and functional richness.

**Species Richness**<br>**Functional Richness**	**2**	**3**	**4**	**6**	**8**	**12**	**15**	**16**
**1**	15							
**2**	14	6						
**3**	13	6	4					
**4**	6	6	18					
**5**	6	8	12	3				
**6**	4	12	6					
**7**	5			9	4			
**8**	4	16						
**9**	4	8	6		9			
**10**	4	8		14	6			
**11**	3			10		5		
**12**	4	6	14		8	4		
**13**	3		15	11		5		
**14**	2	10		8	6	4	2	
**15**	1	10	6	13	3	4	6	1

Table S2. Summary statistics of ABC, ΔABC and the relative biodiversity effect ($\Delta ABC/ABC_{NULL}$).

**Time Period**		**1**	**2**	**3**	**4**
**ABC** [g C/m^2/a]	min	139	-50	-40	-4
	max	206	74	48	2
	Q1	180	55	25	-2
	Q3	192	66	41	1
	mean	185	57	28	-1
**ΔABC** [g C/m^2/a]	min	-5.28	-34.2	-13.9	-1.57
	max	61.3	21.2	27.5	1.41
	Q1	22.8	-8.25	-10.1	-0.691
	Q3	45.9	9.58	-1.68	-0.009
	mean	33.8	0.017	-4.85	-0.351
**$\Delta ABC/ABC_{NULL}$** [%]	min	-3.60	-117	-2750	-248
	max	43.7	45.4	10100	608
	Q1	14.3	-13.0	-24.3	-44.5
	Q3	31.8	16.9	-7.68	12.9
	median	24.0	2.45	-15.5	-8.06

In red: the values that indicate this period to be further analysed for a diversity effect. Q1 and Q3 mark the 1st and 3rd quantiles. Highlighted: relevant criteria for selection.

Table S3. Standardised path coefficients (SPC).

Period 1	F-Ric	F-Diss	F-ID	ΔLAI_{SD}	$\Delta Height_{SD}$	ΔHeight	ΔBM	ΔLAI	ΔWS	ΔM_{Shade}	$\Delta M_{Senescence}$	ΔM_{Storm}	ΔM_{Fire}	$\Delta M_{Crushing}$	ΔGrowth	ΔRecruitment	ΔTurnover	ΔMortality
ΔLAI_{SD}	-0.5	0.4	0.2															
$\Delta Height_{SD}$	1	-0.3	0															
ΔHeight	0.8	0.2	0.5															
ΔBM	0.1	0.1	-0.7															
ΔLAI	-0.4	0	-0.8		0.1	-0.6	0.6											
ΔWS	-0.6	0.1	-0.5		0.1	-0.7	0.7	1.2										
ΔM_{Shade}	-0.2	-0.5	0.2		0	0	-0.9	0										
$\Delta M_{Senescence}$	0.3	0	-0.7				0.3											
ΔM_{Storm}	0.8	0.2	0.4			0.7	0.4											
ΔM_{Fire}	-0.2	0	-0.6		0.1	-0.7	0.7	1.2	1									
$\Delta M_{Crushing}$	-0.5	0	0.5		0	-0.1	-0.2	0		-0.2	-0.9	-0.3						
ΔGrowth	0.4	-0.3	-0.7	-0.8	0.2	-1.2	0.5	1.9	-0.3									
ΔRecruitment	-0.8	0	0.2	0.6	-0.1	0.4	0	-0.6	0.2									
ΔTurnover	-0.4	0	-0.8	-0.1	0.1	-0.7	0.6	1.1										
ΔMortality	-0.2	-0.5	0.1		0	-0.3	-0.9	0.5	0.1	*1*	*0.1*	*0*	*0.1*	*0.1*				
ΔABC	0.6	0.1	-0.3	-0.7	0.1	-0.4	1.2	0.7	-0.4	-1.1	-0.1	0	-0.1	-0.1	*1.1*	*0.3*	*-0.6*	*-1*
**Period 3**	**F-Ric**	**F-Diss**	**F-ID**	**ΔLAI_{SD}**	**$\Delta Height_{SD}$**	**ΔHeight**	**ΔBM**	**ΔLAI**	**ΔWS**	**ΔM_{Shade}**	**$\Delta M_{Senescence}$**	**ΔM_{Storm}**	**ΔM_{Fire}**	**$\Delta M_{Crushing}$**	**ΔGrowth**	**ΔRecruitment**	**ΔTurnover**	**ΔMortality**
ΔLAI_{SD}	0.5	0.7	0.6															
$\Delta Height_{SD}$	0.7	0.6	0.4															
ΔHeight	0.7	0.3	0.4															
ΔBM	0.4	0.1	0.5															
ΔLAI	-0.6	0	0		0.5	-1.7	0.6											
ΔWS	0	0.5	0.6		0.4	-1.3	0.5	0.8										
ΔM_{Shade}	-0.8	-0.2	0		0.3	-1	-0.4	0.6										
$\Delta M_{Senescence}$	0	0.2	0.4				0.9											
ΔM_{Storm}	0.5	0.1	0.6			0.3	0.8											
ΔM_{Fire}	-0.2	0	0.3		0.2	-0.7	0.5	0.4	0.6									
$\Delta M_{Crushing}$	0	0	0.5		0.2	-0.2	0.4	0.3		0.5	-0.1	1						
ΔGrowth	-0.7	-0.2	-0.2	-0.1	0.5	-1.6	0.3	0.9	0.1									
ΔRecruitment	-0.6	-0.6	0.4	-0.2	0.3	-0.9	-0.1	0.5	0.2									
ΔTurnover	-0.6	0	0	0	0.5	-1.7	0.6	1										
ΔMortality	-0.5	0	0.5		0.3	-0.9	0.5	0.6	0.1	*0.9*	*0.6*	*0.4*	*0.1*	*0.3*				
ΔABC	0	-0.2	-0.8	-0.2	0.1	-0.4	-0.4	0.2	0.1	-0.9	-0.6	-0.4	-0.1	-0.3	*1.3*	*0*	*-0.4*	*-1*

Abbreviations: F-Ric = functional richness, F-Diss = functional dissimilarity, F-ID = functional identity, LAI = leaf area index, BM = biomass, WS = water stress, M = Mortality, ABC = annual biomass change. Note that SPC can, in cases of high correlation among variables, also assume values |SPC|>1.0, as they are not equivalent to correlation coefficients (Jöreskog 1999). Table to be read like this: effect from column into row (e.g. SPC in period 1 of F-Ric to ΔABC = 0.6). Shown are only SPC significant at a credible level of 95%. Calculated SPC from mathematical equations appear in italics. Shaded empty fields are invalid combinations that do not occur in the path model.

References

Jöreskog KG (1999) How large can a standardized coefficient be. The help-file of the LISREL program. Available: www.ssicentral.com/lisrel/techdocs/HowLargeCanaStandardizedCoefficientbe.pdf. Accessed March 2014.

Kinzig AP, Pacala SW (2001) Successional biodiversity and ecosystem functioning. In: Kinzig AP, Pacala SW, Tilman D, editors. The functional consequences of biodiversity: empirical progress and theoretical extensions: Princeton University Press. pp. 175–212.

Morin X, Fahse L, Scherer-Lorenzen M, Bugmann H (2011) Tree species richness promotes productivity in temperate forests through strong complementarity between species. Ecology letters 14 (12): 1211–1219.

Wirth C, Lichstein JW (2009) The Imprint of Species Turnover on Old-Growth Forest Carbon Balances-Insights From a Trait-Based Model of Forest Dynamics. In: Wirth C, Gleixner G, Heimann M, editors. Old-Growth Forests. Function, Fate and Value: Springer. pp. 81–113.
