# Supplementary material for: Taking a closer look: disentangling effects of functional diversity on ecosystem functions with a trait-based model across hierarchy and time
Source: R Soc Open Sci. 2015 Mar 25;2(3):140541. doi: 10.1098/rsos.140541 (PMC4448832; doi:10.1098/rsos.140541)
Supplement: Appendix E. Analysis Code and data for path analysis. (PDF) [file rsos140541supp6.pdf]

Appendix E – Analysis Code

Details of path modelling and path analysis

Fitting of the path model (Figure 4, main document) was performed in the program WinBUGS 1.4 (Gilks *et al.* 1994). The parameters controlling the model fit were set to be: number of burn-in iterations: 5'000, number of iterations for posterior distributions: 10'000, number of chains: 4, thinning: 20, such that per chain 500 iterations were saved totaling a number of 2'000 iterations per statistical node in the posterior distributions. We checked convergence for each statistical node by inspecting the history of iterations visually and by requiring that the Gelman-Rubin diagnostic $\hat{R}$ (Gelman and Rubin 1992) be lower than 1.01 for each statistical node, values of $\hat{R} < 1.1$ are deemed acceptable. For each node we provided uninformative flat priors from uniform distributions with a range from -10 to 10 for path coefficients and 0 to 10 for standard errors. Initial values for all statistical nodes were drawn from uniform distributions with a range from 0 to 1. All input data was standardized to a mean of 0 and a standard deviation of 1.

Path analysis (aggregation along pathways) was performed after fitting the model and by sequencing through each iteration thus rendering also posterior distributions for the aggregated paths. These were checked for significance at a credible level of 95% and we reported the means and the credible intervals.

We here report the code for an R-script that reads in the output from the vegetation model (Appendix E in Supplementary Information), performs the path modelling by linking to WinBUGS, performs the path aggregations and writes the results into text files. To run it, it is necessary to adapt the directories and have a copy of WinBUGS14.

References

Gelman A, Rubin DB (1992) Inference from Iterative Simulation using Multiple Sequences. Statistical Science, 7, p. 457–511.

Gilks WR, Thomas A, Spiegelhalter DJ (1994) A language and program for complex Bayesian modelling. The Statistician: 169–177.

R code

```
# Author: Frederic Holzwarth
# Email: frederic.holzwarth@uni-leipzig.de
# Date: December, 2014
# Topic: Path Analysis for aggregated output from LPJ-GUESS

# ========================= #
# === preparatory steps === #
# ========================= #

# ************ #
# *** data *** #
# ************ #

# setting directories
WD <- "D:/Dropbox/lpj guess/Publication/dat/" # input / output directory
bugsdir <- "D:/Program Files/WinBUGS14" # directory of the WinBUGS program
bugswd <- "D:/bugswd"  # working directory for the interaction w/ WinBUGS

# load data
dat0 <- read.csv(paste(WD,"Appendix E data.csv", sep=""), header=T)
# IBM = Increment in BioMass is referred to as "Growth" in the paper
N <- 400
n_nodes <- 80

# split for time periods
dat1 <- dat0[dat0$Period == 1,1:23]
dat3 <- dat0[dat0$Period == 3,1:23]

# scale the variables (z-Transformation)
dat1 <- apply(dat1,2,scale)
dat3 <- apply(dat3,2,scale)

# libraries
library(R2WinBUGS)


# ************ #
# * END data * #
# ************ #


# ************************ #
# *** define functions *** #
# ************************ #

assign_variables <- function(dat=dat1){
  for(i in seq(ncol(dat))){
    x <- dat[,i]
    assign(colnames(dat)[i], x, envir=.GlobalEnv)
    }
}

inits <- function() {
  init_list <- vector("list",n_nodes)
  for(i in seq(n_nodes)){
        init_list[[i]] <- runif(1)
        names(init_list)[i] <- parms[i]
  }
  return(init_list)
}

# ************************ #
# * END define functions * #
# ************************ #

# ========================= #
# = END preparatory steps = #
# ========================= #



# ================================ #
# === Path Analysis in WinBUGS === #
# ================================ #
```

```
# ********************* #
# *** WinBUGS model *** #
# ********************* #

model_ABC <- function(){

  # PRIORS
   R.Re ~ dunif(-10,10)
   R.la ~ dunif(-10,10)
   T.la ~ dunif(-10,10)
   T.ls ~ dunif(-10,10)
   G.la ~ dunif(-10,10)
   G.ls ~ dunif(-10,10)
   G.ws ~ dunif(-10,10)
  Sh.bm ~ dunif(-10,10)
  Sh.la ~ dunif(-10,10)
  Se.bm ~ dunif(-10,10)
  St.bm ~ dunif(-10,10)
  St.hm ~ dunif(-10,10)
  Fi.bm ~ dunif(-10,10)
  Fi.ws ~ dunif(-10,10)
  Cr.Sh ~ dunif(-10,10)
  Cr.Se ~ dunif(-10,10)
  Cr.St ~ dunif(-10,10)
  Ra.G  ~ dunif(-10,10)
  Ra.bm ~ dunif(-10,10)
  la.bm ~ dunif(-10,10)
  la.hm ~ dunif(-10,10)
  la.hs ~ dunif(-10,10)
  ws.la ~ dunif(-10,10)

  Recruitment.prec <- pow( R.sd,-2)
     Turnover.prec <- pow( T.sd,-2)
          GPP.prec <- pow( G.sd,-2)
        Shade.prec <- pow(Sh.sd,-2)
   Senescence.prec <- pow(Se.sd,-2)
        Storm.prec <- pow(St.sd,-2)
         Fire.prec <- pow(Fi.sd,-2)
     Crushing.prec <- pow(Cr.sd,-2)
        RespA.prec <- pow(Ra.sd,-2)
          LAI.prec <- pow(la.sd,-2)
       LAI_SD.prec <- pow(ls.sd,-2)
           WS.prec <- pow(ws.sd,-2)
           BM.prec <- pow(bm.sd,-2)
       height.prec <- pow(hm.sd,-2)
    height_sd.prec <- pow(hs.sd,-2)

   R.sd ~ dunif(0,10)
   T.sd ~ dunif(0,10)
   G.sd ~ dunif(0,10)
  Sh.sd ~ dunif(0,10)
  Se.sd ~ dunif(0,10)
  St.sd ~ dunif(0,10)
  Fi.sd ~ dunif(0,10)
  Cr.sd ~ dunif(0,10)
  Ra.sd ~ dunif(0,10)
  la.sd ~ dunif(0,10)
  ls.sd ~ dunif(0,10)
  ws.sd ~ dunif(0,10)
  bm.sd ~ dunif(0,10)
  hm.sd ~ dunif(0,10)
  hs.sd ~ dunif(0,10)

   r.R  <- 1-pow( R.sd,2)
   r.T  <- 1-pow( T.sd,2)
   r.G  <- 1-pow( G.sd,2)
   r.Sh <- 1-pow(Sh.sd,2)
   r.Se <- 1-pow(Se.sd,2)
   r.St <- 1-pow(St.sd,2)
   r.Fi <- 1-pow(Fi.sd,2)
   r.Cr <- 1-pow(Cr.sd,2)
   r.Ra <- 1-pow(Ra.sd,2)
   r.la <- 1-pow(la.sd,2)
   r.ls <- 1-pow(ls.sd,2)
   r.ws <- 1-pow(ws.sd,2)
   r.bm <- 1-pow(bm.sd,2)
   r.hm <- 1-pow(hm.sd,2)
   r.hs <- 1-pow(hs.sd,2)
```

```
   R.F ~ dunif(-10,10)
   T.F ~ dunif(-10,10)
   G.F ~ dunif(-10,10)
  Sh.F ~ dunif(-10,10)
  Se.F ~ dunif(-10,10)
  St.F ~ dunif(-10,10)
  Fi.F ~ dunif(-10,10)
  Ra.F ~ dunif(-10,10)
  la.F ~ dunif(-10,10)
  ls.F ~ dunif(-10,10)
  ws.F ~ dunif(-10,10)
  bm.F ~ dunif(-10,10)
  hm.F ~ dunif(-10,10)
  hs.F ~ dunif(-10,10)

   R.Q ~ dunif(-10,10)
   T.Q ~ dunif(-10,10)
   G.Q ~ dunif(-10,10)
  Sh.Q ~ dunif(-10,10)
  Se.Q ~ dunif(-10,10)
  St.Q ~ dunif(-10,10)
  Fi.Q ~ dunif(-10,10)
  Ra.Q ~ dunif(-10,10)
  la.Q ~ dunif(-10,10)
  ls.Q ~ dunif(-10,10)
  ws.Q ~ dunif(-10,10)
  bm.Q ~ dunif(-10,10)
  hm.Q ~ dunif(-10,10)
  hs.Q ~ dunif(-10,10)

   R.C ~ dunif(-10,10)
   T.C ~ dunif(-10,10)
   G.C ~ dunif(-10,10)
  Sh.C ~ dunif(-10,10)
  Se.C ~ dunif(-10,10)
  St.C ~ dunif(-10,10)
  Fi.C ~ dunif(-10,10)
  Ra.C ~ dunif(-10,10)
  la.C ~ dunif(-10,10)
  ls.C ~ dunif(-10,10)
  ws.C ~ dunif(-10,10)
  bm.C ~ dunif(-10,10)
  hm.C ~ dunif(-10,10)
  hs.C ~ dunif(-10,10)

  # LIKELIHOOD
  for(i in 1:N){

  Recruitment[i] ~ dnorm(Recruitment.mu[i], Recruitment.prec)
     Turnover[i] ~ dnorm(   Turnover.mu[i],    Turnover.prec)
          GPP[i] ~ dnorm(        GPP.mu[i],         GPP.prec)
        Shade[i] ~ dnorm(      Shade.mu[i],       Shade.prec)
   Senescence[i] ~ dnorm( Senescence.mu[i],  Senescence.prec)
        Storm[i] ~ dnorm(      Storm.mu[i],       Storm.prec)
         Fire[i] ~ dnorm(       Fire.mu[i],        Fire.prec)
     Crushing[i] ~ dnorm(   Crushing.mu[i],    Crushing.prec)
        RespA[i] ~ dnorm(      RespA.mu[i],       RespA.prec)
          LAI[i] ~ dnorm(        LAI.mu[i],         LAI.prec)
       LAI_SD[i] ~ dnorm(     LAI_SD.mu[i],      LAI_SD.prec)
           WS[i] ~ dnorm(         WS.mu[i],          WS.prec)
           BM[i] ~ dnorm(         BM.mu[i],          BM.prec)
       height[i] ~ dnorm(     height.mu[i],      height.prec)
    height_sd[i] ~ dnorm(  height_sd.mu[i],   height_sd.prec)

 Recruitment.mu[i] <- R.F*FRic[i] + R.Q*RaoQ[i] + R.C*CWM[i] + R.la*LAI[i] + R.Re*Reprod[i]
    Turnover.mu[i] <- T.F*FRic[i] + T.Q*RaoQ[i] + T.C*CWM[i] + T.la*LAI[i] + T.ls*LAI_SD[i]
         GPP.mu[i] <- G.F*FRic[i] + G.Q*RaoQ[i] + G.C*CWM[i] + G.la*LAI[i] + G.ls*LAI_SD[i] +
G.ws*WS[i]

    Crushing.mu[i] <- Cr.Sh*Shade[i] + Cr.Se*Senescence[i] + Cr.St*Storm[i]
       Shade.mu[i] <- Sh.F*FRic[i] + Sh.Q*RaoQ[i] + Sh.C*CWM[i] + Sh.bm*BM[i] + Sh.la*LAI[i]
  Senescence.mu[i] <- Se.F*FRic[i] + Se.Q*RaoQ[i] + Se.C*CWM[i] + Se.bm*BM[i]
       Storm.mu[i] <- St.F*FRic[i] + St.Q*RaoQ[i] + St.C*CWM[i] + St.bm*BM[i] +
St.hm*height[i]
        Fire.mu[i] <- Fi.F*FRic[i] + Fi.Q*RaoQ[i] + Fi.C*CWM[i] + Fi.bm*BM[i] + Fi.ws*WS[i]

       RespA.mu[i] <- Ra.F*FRic[i] + Ra.Q*RaoQ[i] + Ra.C*CWM[i] + Ra.bm*BM[i] + Ra.G*GPP[i]
```

```
        LAI.mu[i] <- la.F*FRic[i] + la.Q*RaoQ[i] + la.C*CWM[i] + la.bm*BM[i] +
la.hm*height[i] + la.hs*height_sd[i]
         WS.mu[i] <- ws.F*FRic[i] + ws.Q*RaoQ[i] + ws.C*CWM[i] + ws.la*LAI[i]

      LAI_SD.mu[i] <- ls.F*FRic[i] + ls.Q*RaoQ[i] + ls.C*CWM[i]
          BM.mu[i] <- bm.F*FRic[i] + bm.Q*RaoQ[i] + bm.C*CWM[i]
      height.mu[i] <- hm.F*FRic[i] + hm.Q*RaoQ[i] + hm.C*CWM[i]
   height_sd.mu[i] <- hs.F*FRic[i] + hs.Q*RaoQ[i] + hs.C*CWM[i]
  }

}
# write the model file
setwd(bugswd)
write.model(model_ABC,"model_ABC.txt")

# BUGS INPUT

data <- c("Recruitment","Turnover","Reprod","GPP","RespA",
          "Shade","Senescence","Storm","Fire","Crushing",
          "RespA","WS","BM","LAI","LAI_SD","height","height_sd",
          "FRic","RaoQ","CWM","N")
parms <- c("G.F","G.Q","G.C","G.la","G.ls","G.ws","G.sd",
           "R.F","R.Q","R.C","R.Re","R.la","R.sd",
           "T.F","T.Q","T.C","T.la","T.ls","T.sd",
           "Cr.Sh","Cr.Se","Cr.St","Cr.sd",
           "Sh.F","Sh.Q","Sh.C","Sh.bm","Sh.la","Sh.sd",
           "Se.F","Se.Q","Se.C","Se.bm",        "Se.sd",
           "St.F","St.Q","St.C","St.bm","St.hm","St.sd",
           "Fi.F","Fi.Q","Fi.C","Fi.bm","Fi.ws","Fi.sd",
           "ws.F","ws.Q","ws.C","ws.la","ws.sd",
           "Ra.F","Ra.Q","Ra.C","Ra.G", "Ra.bm","Ra.sd",
           "la.F","la.Q","la.C","la.bm","la.hm","la.hs","la.sd",
           "ls.F","ls.Q","ls.C","ls.sd",
           "bm.F","bm.Q","bm.C","bm.sd",
           "hm.F","hm.Q","hm.C","hm.sd",
           "hs.F","hs.Q","hs.C","hs.sd",

           "r.G","r.R","r.T","r.Sh","r.Se","r.St","r.Fi","r.Cr",
           "r.Ra","r.la","r.ls","r.ws","r.bm","r.hm","r.hs")

unlist(inits()) # just a check

# ~~~~~~~~~~~~~~~~~~~~~~~~~ #
# ~~~ time periods loop ~~~ #
# ~~~~~~~~~~~~~~~~~~~~~~~~~ #

for (time_period in c(1,3)){
  assign_variables(get(paste("dat",time_period,sep="")))
  # time_period <- 1; assign_variables(dat1) # or by hand

  bugs_out <- bugs(data, inits, parms, model="model_ABC.txt",
               n.iter=15000, n.burnin=5000, n.thin=20, n.chain=4,
               bugs.dir=bugsdir, working.dir=bugswd, debug=F, DIC=T, save.history=T)
               # set debug = TRUE to remain inside WinBUGS

  assign(paste("bugs_out_",time_period,sep=""),bugs_out)
}

# ~~~~~~~~~~~~~~~~~~~~~~~~~ #
# ~ END time periods loop ~ #
# ~~~~~~~~~~~~~~~~~~~~~~~~~ #

print(bugs_out_1,2)
print(bugs_out_3,2)
# save output
# save(file=paste(WD,"bugs_out.Rdata",sep=""),bugs_out_1,bugs_out_3)
# load(paste(WD,"bugs_out.Rdata",sep=""))

# max(Rhat) for each time period
# Rhat = Gelman-Rubin diagnostic of convergence, should be < 1.1
max(bugs_out_1$summary[,8]) # 1.005328
max(bugs_out_3$summary[,8]) # 1.006059

# ********************* #
# * END WinBUGS model * #
# ********************* #
```

```r
# ********************* #
# *** path analysis *** #
# ********************* #

# important to have the correct name sequence
ABC_names_seq <-
c("FRic","RaoQ","CWM","LAI_SD","Height_SD","Height","BM","LAI","WS","Shade","Senescence","Stor
m","Fire","Crushing","GPP","RespA","NPP","Reprod.","IBM","Recruitment","Turnover","Mortality",
"ABC")


# ~~~~~~~~~~~~~~~~~~~~~~~~~ #
# ~~~ time periods loop ~~~ #
# ~~~~~~~~~~~~~~~~~~~~~~~~~ #

for (time_period in c(1,3)){
  assign_variables(get(paste("dat",time_period,sep="")))
  # time_period <- 1; assign_variables(dat1) # or by hand
  # time_period <- 3; assign_variables(dat3) # or by hand

  obj_bugs <- get(paste("bugs_out_",time_period,sep=""))

  # assign calculated parameters
  ABCV <- coef(lm(ABC ~ 0 + IBM + Recruitment + Turnover + Mortality))
  IBMV <- c(coef(lm(Reprod ~ 0 + NPP)),coef(lm(IBM ~ 0 + NPP + Reprod)))
  NPPV <- coef(lm(NPP ~ 0 + GPP + RespA))
  MortalityV <- coef(lm(Mortality ~ 0 + Shade + Senescence + Storm + Fire + Crushing))
  ABCV;IBMV;NPPV;MortalityV

  A.I  <- ABCV[1]
  A.R  <- ABCV[2]
  A.T  <- ABCV[3]
  A.M  <- ABCV[4]
  Re.N <- IBMV[1]
  I.N  <- IBMV[2]
  I.Re <- IBMV[3]
  N.G  <- NPPV[1]
  N.Ra <- NPPV[2]
  M.Sh <- MortalityV[1]
  M.Se <- MortalityV[2]
  M.St <- MortalityV[3]
  M.Fi <- MortalityV[4]
  M.Cr <- MortalityV[5]

  # prepare matrix of path coefficients (SPC)
  n_parm <- 23
  n_sims <- obj_bugs$n.sims

  # a matrix for single SPC estimates
  T0 <- diag(n_parm)
  colnames(T0) <- ABC_names_seq
  rownames(T0) <- ABC_names_seq

  # matrices for all iterations
  T1 <- array(dim=c(n_parm,n_parm,n_sims))

  # loop through simulations
  for(k in seq(n_sims)){

    # get the values for the nodes
    for (j in seq(n_nodes))
      assign(names(obj_bugs$sims.list[j]),obj_bugs$sims.matrix[k,j])

    # use them for PA
    for(i in seq(n_parm)){
      if(any(T0[i,1:3] != 0)){
        T0[i,4]  <- ls.F*T0[i,1] + ls.Q*T0[i,2] + ls.C*T0[i,3] # lAI_SD    4
        T0[i,5]  <- hs.F*T0[i,1] + hs.Q*T0[i,2] + hs.C*T0[i,3] # height_SD 5
        T0[i,6]  <- hm.F*T0[i,1] + hm.Q*T0[i,2] + hm.C*T0[i,3] # height    6
        T0[i,7]  <- bm.F*T0[i,1] + bm.Q*T0[i,2] + bm.C*T0[i,3] # BM        7
      }
      if(any(T0[i,c(1:3,5:7)] != 0)) # LAI 8
        T0[i,8]  <- la.F*T0[i,1] + la.Q*T0[i,2] + la.C*T0[i,3] + la.bm*T0[i,7] + la.hm*T0[i,6]
+ la.hs*T0[i,5]
      if(any(T0[i,c(1:3,8)] != 0))   # WS  9
        T0[i,9]  <- ws.F*T0[i,1] + ws.Q*T0[i,2] + ws.C*T0[i,3] + ws.la*T0[i,8]

      if(any(T0[i,c(1:3,7,8)] != 0)) # Shade      10
```

```
        T0[i,10] <- Sh.F*T0[i,1] + Sh.Q*T0[i,2] + Sh.C*T0[i,3] + Sh.bm*T0[i,7] + Sh.la*T0[i,8]
      if(any(T0[i,c(1:3,7)] != 0))   # Senescence 11
        T0[i,11] <- Se.F*T0[i,1] + Se.Q*T0[i,2] + Se.C*T0[i,3] + Se.bm*T0[i,7]
      if(any(T0[i,c(1:3,7,6)] != 0)) # Storm      12
        T0[i,12] <- St.F*T0[i,1] + St.Q*T0[i,2] + St.C*T0[i,3] + St.bm*T0[i,7] + St.hm*T0[i,6]
      if(any(T0[i,c(1:3,7,9)] != 0)) # Fire       13
        T0[i,13] <- Fi.F*T0[i,1] + Fi.Q*T0[i,2] + Fi.C*T0[i,3] + Fi.bm*T0[i,7] + Fi.ws*T0[i,9]
      if(any(T0[i,c(10:12)] != 0))   # Crushing   14
        T0[i,14] <- Cr.Sh*T0[i,10] + Cr.Se*T0[i,11] + Cr.St*T0[i,12]

      if(any(T0[i,c(1:3,4,8,9)] != 0)) # GPP          15
        T0[i,15] <- G.F*T0[i,1] + G.Q*T0[i,2] + G.C*T0[i,3] + G.la*T0[i,8] + G.ls*T0[i,4] +
G.ws*T0[i,9]
      if(any(T0[i,c(1:3,7,15)] != 0))  # RespA        16
        T0[i,16] <- Ra.F*T0[i,1] + Ra.Q*T0[i,2] + Ra.C*T0[i,3] + Ra.G*T0[i,15] + Ra.bm*T0[i,7]
      if(any(T0[i,c(15:16)] != 0))     # NPP          17
        T0[i,17] <- N.G*T0[i,15] + N.Ra*T0[i,16]
      if(any(T0[i,c(17)] != 0))        # Reproduction 18
        T0[i,18] <- Re.N*T0[i,17]
      if(any(T0[i,c(17:18)] != 0))     # IBM          19
        T0[i,19] <- I.N*T0[i,17] + I.Re*T0[i,18]
      if(any(T0[i,c(1:3,8,18)] != 0))  # Recruitment  20
        T0[i,20] <-  R.F*T0[i,1] + R.Q*T0[i,2] + R.C*T0[i,3] + R.la*T0[i,8] + R.Re*T0[i,18]
      if(any(T0[i,c(1:3,4,8)] != 0))   # Turnover     21
        T0[i,21] <-  T.F*T0[i,1] + T.Q*T0[i,2] + T.C*T0[i,3] + T.la*T0[i,8] + T.ls*T0[i,4]
      if(any(T0[i,c(10:14)] != 0))     # Mortality    22
        T0[i,22] <- M.Sh*T0[i,10] + M.Se*T0[i,11] + M.St*T0[i,12] + M.Fi*T0[i,13] +
M.Cr*T0[i,14]
                                       # ABC          23
        T0[i,23] <- A.I*T0[i,19] + A.R*T0[i,20] + A.T*T0[i,21] + A.M*T0[i,22]
      }
    T1[,,k] <- T0
    if(!(k  100)) print(k)
  }

  assign(paste("T1_",time_period,sep=""),T1)

}

# ~~~~~~~~~~~~~~~~~~~~~~~~~ #
# ~ END time periods loop ~ #
# ~~~~~~~~~~~~~~~~~~~~~~~~~ #

str(T1_1)
str(T1_3)
round(apply(T1_1,1:2,mean),1)

# ******************** #
# * END path analysis * #
# ******************** #



# *********************** #
# *** path aggregation *** #
# *********************** #

# ~~~~~~~~~~~~~~~~~~~~~~~~~ #
# ~~~ time periods loop ~~~ #
# ~~~~~~~~~~~~~~~~~~~~~~~~~ #

for (time_period in c(1,3)){
  T2 <- get(paste("T1_",time_period,sep=""))
  # or by hand
  # time_period <- 1; T2 <- T1_1
  # time_period <- 3; T2 <- T1_3


  # ~~~ credible intervals ~~~ #

  alpha <- 0.05 # for 95% credible interval

  T1_mean <- apply(T2,1:2,mean)
  T1_hi <- apply(T2,1:2,quantile,p=1-alpha/2)
  T1_lo <- apply(T2,1:2,quantile,p=alpha/2)

  colnames(T1_mean) <- ABC_names_seq
  rownames(T1_mean) <- ABC_names_seq
```

```
# whether singificant at 1-alpha
T1_sign <- sign(T1_hi) == sign(T1_lo)
T1_mean_sign <- T1_mean*T1_sign
assign(paste("T1_mean",time_period,sep=""),T1_mean)
assign(paste("T1_mean_sign",time_period,sep=""),T1_mean_sign)


# ~~~~~~~~~~~~~~~~~~~~ #
# ~~~ FC-RATES-ABC ~~~ #
# ~~~~~~~~~~~~~~~~~~~~ #

which_rates <- 19:22
T3 <- T2[1:3,1:4,]*NA
for(i in seq(n_sims))
  T3[,,i] <- t(t(T2[1:3,which_rates,i]) * T2[which_rates,23,i])

T3_mean <- apply(T3,1:2,mean)
T3_hi <- apply(T3,1:2,quantile,p=1-alpha/2)
T3_lo <- apply(T3,1:2,quantile,p=alpha/2)

colnames(T3_mean) <- paste("ABC",ABC_names_seq[which_rates],sep="_")
rownames(T3_mean) <- ABC_names_seq[1:3]
# whether singificant at 1-alpha
T3_sign <- sign(T3_hi) == sign(T3_lo)
T3_mean_sign <- T3_mean*T3_sign
assign(paste("T3_mean",time_period,sep=""),T3_mean)
assign(paste("T3_mean_sign",time_period,sep=""),T3_mean_sign)

# ~~~~~~~~~~~~~~~~~~~~ #
# ~ END FC-RATES-ABC ~ #
# ~~~~~~~~~~~~~~~~~~~~ #


# ~~~~~~~~~~~~~~~~~~~~~ #
# ~~~ FC-STATES-ABC ~~~ #
# ~~~~~~~~~~~~~~~~~~~~~ #

which_states <- 4:9
T4 <- T2[1:3,1:6,]*NA
for(i in seq(n_sims))
  T4[,,i] <- t(t(T2[1:3,which_states,i]) * T2[which_states,23,i])

T4_mean <- apply(T4,1:2,mean)
T4_hi <- apply(T4,1:2,quantile,p=1-alpha/2)
T4_lo <- apply(T4,1:2,quantile,p=alpha/2)

colnames(T4_mean) <- paste("ABC",ABC_names_seq[which_states],sep="_")
rownames(T4_mean) <- ABC_names_seq[1:3]
# whether singificant at 1-alpha
T4_sign <- sign(T4_hi) == sign(T4_lo)
T4_mean_sign <- T4_mean*T4_sign
assign(paste("T4_mean",time_period,sep=""),T4_mean)
assign(paste("T4_mean_sign",time_period,sep=""),T4_mean_sign)

# ~~~~~~~~~~~~~~~~~~~~~ #
# ~ END FC-STATES-ABC ~ #
# ~~~~~~~~~~~~~~~~~~~~~ #


# ~~~~~~~~~~~~~~~~~~~~~~~~~ #
# ~~~ FC-MORT-RATES-ABC ~~~ #
# ~~~~~~~~~~~~~~~~~~~~~~~~~ #

which_mort <- 10:14
T5 <- T2[1:3,1:5,]*NA
for(i in seq(n_sims))
  T5[,,i] <- t(t(T2[1:3,which_mort,i]) * T2[which_mort,23,i])

T5_mean <- apply(T5,1:2,mean)
T5_hi <- apply(T5,1:2,quantile,p=1-alpha/2)
T5_lo <- apply(T5,1:2,quantile,p=alpha/2)

colnames(T5_mean) <- paste("ABC",ABC_names_seq[which_mort],sep="_")
rownames(T5_mean) <- ABC_names_seq[1:3]
# whether singificant at 1-alpha
T5_sign <- sign(T5_hi) == sign(T5_lo)
T5_mean_sign <- T5_mean*T5_sign
assign(paste("T5_mean",time_period,sep=""),T5_mean)
```

```r
  assign(paste("T5_mean_sign",time_period,sep=""),T5_mean_sign)

  # ~~~~~~~~~~~~~~~~~~~~~~~~~ #
  # ~ END FC-MORT-RATES-ABC ~ #
  # ~~~~~~~~~~~~~~~~~~~~~~~~~ #

}
# ~~~~~~~~~~~~~~~~~~~~~~~~~ #
# ~ END time periods loop ~ #
# ~~~~~~~~~~~~~~~~~~~~~~~~~ #

# ************************ #
# * END path aggregation * #
# ************************ #

# ================================ #
# = END Path Analysis in WinBUGS = #
# ================================ #



# ****************** #
# *** data output *** #
# ****************** #

# ~~~~~~~~~~~~~~~~~~ #
# ~~~ model fits ~~~ #
# ~~~~~~~~~~~~~~~~~~ #

r2_seq <- setdiff(grep("r.",names(bugs_out_1$mean)),grep("Cr.",names(bugs_out_1$mean)))
r2_tab_raw <- cbind(unlist(bugs_out_1$mean[r2_seq]),unlist(bugs_out_3$mean[r2_seq]))
r2_tab <- round(r2_tab_raw,2)
r2_tab
write.table(r2_tab,file=paste(WD,"r2_tab.txt",sep=""), quote=F, sep="\t")

# ~~~~~~~~~~~~~~~~~~ #
# ~ END model fits ~ #
# ~~~~~~~~~~~~~~~~~~ #

T1a <- round(t(T1_mean_sign1),1) - diag(n_parm)
T3a <- round(t(T1_mean_sign3),1) - diag(n_parm)
T1a3 <- round(t(T1_mean_sign1),3) - diag(n_parm)
T3a3 <- round(t(T1_mean_sign3),3) - diag(n_parm)

T1c <- round(t(T3_mean_sign1),1)
T3c <- round(t(T3_mean_sign3),1)
T1c3 <- round(t(T3_mean_sign1),3)
T3c3 <- round(t(T3_mean_sign3),3)

T1d <- round(t(T4_mean_sign1),1)
T3d <- round(t(T4_mean_sign3),1)
T1d3 <- round(t(T4_mean_sign1),3)
T3d3 <- round(t(T4_mean_sign3),3)

T1e <- round(t(T5_mean_sign1),1)
T3e <- round(t(T5_mean_sign3),1)
T1e3 <- round(t(T5_mean_sign1),3)
T3e3 <- round(t(T5_mean_sign3),3)

write.table(T1a,file=paste(WD,"T1a.txt",sep=""), quote=F, sep="\t")
write.table(T3a,file=paste(WD,"T3a.txt",sep=""), quote=F, sep="\t")
write.table(T1a3,file=paste(WD,"T1a3.txt",sep=""), quote=F, sep="\t")
write.table(T3a3,file=paste(WD,"T3a3.txt",sep=""), quote=F, sep="\t")

write.table(cbind(T1c,T3c),file=paste(WD,"T13c.txt",sep=""), quote=F, sep="\t")
write.table(cbind(T1d,T3d),file=paste(WD,"T13d.txt",sep=""), quote=F, sep="\t")
write.table(cbind(T1e,T3e),file=paste(WD,"T13e.txt",sep=""), quote=F, sep="\t")

write.table(cbind(T1c3,T3c3),file=paste(WD,"T13c3.txt",sep=""), quote=F, sep="\t")
write.table(cbind(T1d3,T3d3),file=paste(WD,"T13d3.txt",sep=""), quote=F, sep="\t")
write.table(cbind(T1e3,T3e3),file=paste(WD,"T13e3.txt",sep=""), quote=F, sep="\t")

# ****************** #
# * END data output * #
# ****************** #

1.25^.5+.5
# EOF
```
